# Development and Evaluation of a Dry Emulsion of Ostrich Oil as a Dietary Supplement

**DOI:** 10.3390/foods13162570

**Published:** 2024-08-17

**Authors:** Juthaporn Ponphaiboon, Sontaya Limmatvapirat, Chutima Limmatvapirat

**Affiliations:** 1 Department of Industrial Pharmacy, Faculty of Pharmacy, Silpakorn University, Nakhon Pathom 73000, Thailand; augusto_sc@hotmail.co.th (J.P.); limmatvapirat_s@su.ac.th (S.L.); 2 Natural Products Research Center (NPRC), Faculty of Pharmacy, Silpakorn University, Nakhon Pathom 73000, Thailand

**Keywords:** ostrich oil, dry emulsion, emulsifier, adsorbent, antioxidant, stability

## Abstract

This study aimed to develop a high-quality dry emulsion incorporating omega-3, 6, and 9 fatty acid-rich ostrich oil for use as a dietary supplement. Extracted from abdominal adipose tissues using a low-temperature wet rendering method, the ostrich oil exhibited antioxidant properties, favorable physicochemical properties, microbial counts, heavy metal levels, and fatty acid compositions, positioning it as a suitable candidate for an oil-in-water emulsion and subsequent formulation as a dry emulsion. Lecithin was employed as the emulsifier due to its safety and health benefits. The resulting emulsion, comprising 10% *w*/*w* lecithin and 10% *w*/*w* ostrich oil, was stable, with a droplet size of 3.93 ± 0.11 μm. This liquid emulsion underwent transformation into a dry emulsion to preserve the physicochemical stability of ostrich oil, utilizing Avicel^®^ PH-101 or Aerosil^®^ 200 through a granulation process. Although Aerosil^®^ 200 exhibited superior adsorption, Avicel^®^ PH-101 granules surpassed it in releasing the ostrich oil emulsion. Consequently, Avicel^®^ PH-101 was selected as the preferred adsorbent for formulating the ostrich oil dry emulsion. The dry emulsion, encapsulated with a disintegration time of 3.11 ± 0.14 min for ease of swallowing, maintained microbial loads and heavy metal contents within acceptable limits. Presented as granules containing butylated hydroxytoluene, the dry emulsion showcased robust temperature stability, suggesting the potential incorporation of animal fat into dry emulsions as a promising dietary supplement.

## 1. Introduction

Ostrich (*Struthio camelus*), a member of the ratite family, produces oil derived from the adipose tissues found in the abdominal cavities and subcutaneous areas of its breasts and back. This oil, known for its rich properties, can serve as an active ingredient in cosmetics [1]. Ostrich oil is prepared through dry or wet rendering, with low-temperature rendering preferred to preserve heat-sensitive compounds like essential fatty acids. High-quality oil is pale to golden yellow, with a smooth texture, medium viscosity, and a subtle, nearly odorless scent [2]. Fats and oils’ stability primarily hinges on rancidity, a critical quality parameter. Rancidity arises from chemical changes, including hydrolysis and oxidation, leading to undesirable odors and flavors [3]. Hydrolytic rancidity results from the hydrolysis of triacylglycerols by lipase and water, while oxidative rancidity involves the degradation of unsaturated fatty acids by oxygen. Oil quality is assessed through peroxide, acid, iodine, and saponification values. Assessing heavy metal content with inductively coupled plasma-mass spectrometry (ICP-MS) is crucial for evaluating health risks and ensuring the product’s quality, safety, and nutritional value [4]. Hence, a primary challenge in formulating products with ostrich oil is enhancing their stability and safety. Ostrich oil is predominantly composed of triacylglycerols and essential fatty acids, with a notable presence of oleic acid (omega-9), linoleic acid (omega-6), and α-linolenic acid (omega-3). Since the human body cannot synthesize omega-3 fatty acids autonomously, their inclusion in the diet is imperative for maintaining good health [5]. These fatty acids confer various health benefits, such as regulating normal metabolism, enhancing heart health, preventing cardiovascular diseases, and influencing brain function and the nervous system [6,7].

Recent advancements in the formulation of ostrich oil-based emulsions have highlighted their potential for enhancing health and skincare applications. Research has demonstrated that nanoemulsions incorporating ostrich oil exhibit significant anti-inflammatory activity. For instance, a nanoemulsion with 1% *w*/*w* ostrich oil, stabilized using Tween-80 and Labrasol, showed notable anti-inflammatory effects in rat models [8]. This study underscores the nanoemulsion’s capability for topical delivery, enhancing the anti-inflammatory properties of ostrich oil. Additionally, the development of emulsions enriched with high concentrations of ostrich oil (20% *w*/*w*) has been explored for skincare applications. This formulation leverages the high oleic acid content of ostrich oil and utilizes Span and Tween as emulsifiers [9]. Research into this area elucidates the role of effective emulsification in optimizing both the efficacy and stability of ostrich oil in skincare products, emphasizing its importance for practical applications where stability and performance are critical. These studies collectively advance our understanding of how ostrich oil can be effectively incorporated into various emulsion systems to enhance its therapeutic and cosmetic benefits. However, there is a notable gap in the development of dry emulsion formulations containing high levels of ostrich oil for use as dietary supplements. Addressing this gap is crucial for expanding the applications of ostrich oil in health care and maximizing its potential benefits.

Edible oils are commonly formulated as oil-in-water (O/W) emulsions to reduce their greasy appearance and enhance palatability and acceptability in food products. O/W emulsions also mask the rancid taste of oil, improving the overall sensory profile. This research focused on formulating edible O/W emulsions containing ostrich oil. Emulsifiers like lecithin, polysorbates, and mono- and diglycerides are essential for stabilizing emulsions by reducing surface tension between oil and water. They form a protective film around oil droplets, preventing flocculation and coalescence. Emulsifiers must be edible, odorless, and tasteless for oral preparations. Soy lecithin [10], derived from soybean oil, is widely used for its molecular structure that allows effective interaction with both oil and water phases. Its low hydrophilic–lipophilic balance (HLB) value makes it particularly effective for stabilizing O/W emulsions. In this study, soy lecithin was combined with the phase inversion technique to achieve stable O/W emulsions with finely dispersed oil droplets, optimizing stability and droplet size.

Dry emulsions are developed to overcome the physicochemical and compliance challenges of liquid emulsions. By removing water, they enhance stability, minimize chemical reactions, and prevent physical instability like phase separation. This water-free environment also protects active ingredients from light, heat, and oxygen, prolonging shelf life and preserving efficacy. Additionally, dry emulsions improve consumer convenience by eliminating the need to measure and handle liquids, boosting user-friendliness and compliance [11,12,13].

In this study, our objective was to prepare a stable and efficient dry emulsion using a simple method with ostrich oil, suitable for application in the pharmaceutical industry. The simple preparation of dry emulsions is achieved by converting liquid O/W emulsions into dry powders through techniques that involve adsorption on solid carriers or adsorbents. Careful selection of edible adsorbents is crucial for dry emulsion preparation. Avicel^®^ PH-101 (FMC BioPolymer, Philadelphia, PA, USA), a tasteless, odorless microcrystalline cellulose, serves as a versatile adsorbent with high water absorption and rapid disintegration, offering advantages in wet granulation formulations such as enhanced drug content uniformity and tablet hardness [14]. Aerosil^®^ 200 (FMC BioPolymer, Philadelphia, PA, USA), a hydrophilic fumed silica, is recognized for its multifunctionality, acting as an effective adsorbent, thickening agent, and anti-caking agent. In dry emulsions, it stabilizes components during drying, enhances flowability, and functions as a rheology control agent [15]. The careful selection of adsorbents underscores their significant contributions to the formulation and stability of dry emulsions. Numerous studies have demonstrated that incorporating edible oils in O/W emulsions administered orally can enhance the absorption and bioavailability of poorly water-soluble oils [16]. Solid oral dosage forms like capsules, known for their ease of swallowing, are preferred. Therefore, the resulting ostrich oil dry emulsions should be filled into hard gelatin capsules. Furthermore, liberation of ostrich oil from the absorbents, reconstitution of dry emulsions, and disintegration time of capsules containing dry emulsion were performed.

Ostrich oil is prone to oxidative and hydrolytic rancidity, resulting in undesirable odors and flavors [17]. No research has focused on developing a dry emulsion dietary supplement with high levels of ostrich oil. Such a supplement should be portable, stable, and produced through a simple process, serving as a substitute to address essential fatty acid deficiencies, particularly omega fatty acids. To tackle this, O/W emulsions with ostrich oil were created, using emulsifiers to protect oil droplets from oxygen and light exposure. Lecithin, an emulsifier known for its benefits to liver, heart, and brain functions [9], was chosen for its ability to create stable emulsions for dry emulsion production. Dry emulsions, derived from liquid O/W emulsions, effectively deliver lipophilic and sensitive compounds while addressing physicochemical and microbial instability. This was achieved by adsorbing the liquid emulsion onto adsorbents like Avicel^®^ PH-101 and Aerosil^®^ 200, forming lipid-based granules that can be reconstituted into O/W emulsions and encapsulated in hard gelatin capsules. The process ensures enhanced oil stability and effective release of the emulsion powder upon oral administration [18]. The research aimed to evaluate the feasibility of manufacturing stable dietary supplements with ostrich oil using dry emulsions.

## 2. Materials and Methods

### 2.1. Materials

All reagents utilized in the experiments were of analytical reagent (AR) and American Chemical Society (ACS) grade. Methyl heptadecanoate and all fatty acid GC standards were procured from Nu-Chek Prep, Inc., Elysian, MN, USA. The ICP multi-element standard solution XIII was acquired from Agilent Technologies, Santa Clara, CA, USA. Purchased from Sigma-Aldrich, Saint Louis, MO, USA: 2,2-diphenyl-1-picrylhydrazyl (DPPH), 6-hydroxy-2,5,7,8-tetramethylchroman-2-carboxylic acid (Trolox), ethylenediaminetetraacetic acid (EDTA), and Wijs solution. Soy lecithin was bought from Tianjin Hexiyuan Lecithin Technology Co., Ltd., Tianjin, China. Avicel^®^ PH-101 and Aerosil^®^ 200 were bought from FMC BioPolymer, Philadelphia, PA, USA, and Evonik Resource Efficiency GmbH, Hanau-Wolfgang, Germany, respectively.

### 2.2. Preparation and Evaluation of Ostrich Oil

#### 2.2.1. Preparation of Ostrich Oil

Frozen abdominal ostrich fat was bought from Siam Ostrich Co., Ltd., situated in Bosuphan, Song Phi Nong district, Suphan Buri 72110, Thailand. Following thawing, the fat was meticulously dried with tissue paper, cut into small pieces, and finely chopped into a smooth paste. In line with a previous report [19], the rendering method for the ostrich paste was conducted at a low temperature of 45–50 °C, with continuous stirring until complete melting was achieved. Subsequently, liquid ostrich oil was carefully separated from any unmelted residues. The resulting ostrich oil was then securely stored in nitrogen-filled amber glass vials at 4 °C, shielded from light, until it underwent analysis and was ready for formulation.

#### 2.2.2. Fatty Acid Composition 

The synthesis of fatty acid methyl esters (FAMEs) and the GC-FID method are detailed in a previous report [19]. FAMEs were produced through a one-step extraction and direct transesterification process involving ostrich oil containing fatty acids and a mixture of methanol: sulfuric acid: chloroform (17: 3: 20, *v*/*v*/*v*). This reaction occurred at 90 °C for 30 min in a heat block (AccuBlockTM Digital Dry Bath, Labnet International, Inc., Edison, NJ, USA). Analysis of FAMEs was conducted using GC-FID with an automated liquid sampler (Model 6890N Network GC System, Agilent Technologies, Santa Clara, CA, USA) and a capillary fused silica column HP-INNOWax 19091N-113 (30 m × 0.32 mm × 0.25 μm film of polyethylene glycol) from Agilent Technologies, Santa Clara, CA, USA. Methyl heptadecanoate served as the internal standard. The GC oven program included an initial temperature of 180 °C, a hold time of 20 min, a rate of 10 °C/min to 240 °C, and a hold time of 4 min. The split ratio was 80:1, with an injection volume of 1 µL. The column flow (He) was 1.5 mL/min in a constant flow mode. For FID, the heater temperature was 300 °C, H_2_ flow was 30 mL/min, air flow was 300 mL/min, and makeup flow (N_2_) was 30 mL/min. Peak identification and quantification of fatty acids were achieved by directly comparing peak areas and retention times of FAMEs in oil samples with those obtained from FAME mix standards.

#### 2.2.3. Antioxidant Activity

##### DPPH Radical Scavenging

The assessment of a sample’s DPPH radical scavenging capacity, reflecting its ability to neutralize DPPH radicals by donating hydrogen atoms or electrons, followed the procedures outlined in our previous studies [20,21]. DPPH radicals, characterized by a deep violet color, display a prominent absorption band at 517 nm. The reduction in DPPH radicals (violet color) to the diamagnetic DPPH (yellow color) in the presence of antioxidants leads to a decrease in absorbance. For the analysis, samples were individually dissolved in ethyl acetate to attain various test concentrations. Following this, 1.0 mL of the sample solution was mixed with 3.0 mL of freshly prepared DPPH solution (0.1 mmol/L) in ethyl acetate and incubated at room temperature for 90 min in darkness. The absorbance was promptly measured against a blank using a UV–vis spectrophotometer (Hitachi U-2900, Hitachi High-Technologies Corporation, Tokyo, Japan). Trolox, the standard antioxidant, was dissolved in ethyl acetate. The 50 percent inhibitory concentration (IC_50_) was determined through linear regression.

##### Copper-Chelating Activity

The capacity of the oil samples to chelate pro-oxidative Cu^2+^ ions was investigated following a previously established protocol [22]. In the experimental procedure, 1 mL of 2 mmol/L CuSO_4_ was combined with 1 mL of pyridine (pH 7.0) and 20 µL of 0.1% *w*/*v* pyrocatechol violet. Subsequently, upon adding 1 mL of the sample solution, the fading of the blue color, indicative of Cu^2+^ dissociation, was monitored by measuring the absorbance at 632 nm over 5 min. A blank was prepared using an equivalent volume of distilled water instead of the sample. EDTA served as a positive control, and the Cu^2+^ chelating activity was subsequently calculated.

#### 2.2.4. Acid Value (AV), Peroxide Value (PV), Iodine Value (IV), Saponification Value (SV), and Refractive Index (RI)

The assessment of AV, PV, IV, and SV involved the use of standard solutions. Potassium hydroxide was employed to neutralize free acids in the sample for AV. Sodium thiosulfate was used to titrate liberated iodine from potassium iodide for PV and to titrate iodine present in the sample, which had reacted with Wijs solution and potassium iodide, for IV. Hydrochloric acid was utilized to back-titrate the excess potassium hydroxide for SV. These standard solutions underwent titration using a potentiometric autotitrator, Titrino Plus 848 (Metrohm, Herisau, Switzerland), following the official procedures outlined by the American Oil Chemists’ Society (AOCS) in Cd 3d-63 [23], Cd 8b-90 [24], Cd 1d-92 [25], and Cd 3-25 [26].

The RI of ostrich oil was measured at 30 ± 0.1 °C using a digital refractometer, NAR-1T Liquid (Atago Co., Ltd., Tokyo, Japan). Prior to obtaining measurements, the instrument was calibrated by adjusting the compensator dial.

#### 2.2.5. Heavy Metal Contents

The concentrations of heavy metals were determined through ICP-MS using a 7500 ce instrument (Agilent Technologies, CA, USA), following the methodology outlined in our prior report with some modifications [27]. Each sample, weighing approximately 0.3 g, underwent digestion by adding 7.0 mL of a 60% *v*/*v* nitric acid solution for 45 min using a microwave digester (Model ETHOS ONE, Milestone Corporation, Sorisole, Italy). The resulting digestate was then diluted with ultrapure water (ASTM type I, 18.3 MΩ × cm resistivity) before analysis by ICP-MS.

#### 2.2.6. Microbial Contamination

The assessment of microbial contamination included analyzing the total aerobic microbial count (TAMC), the total combined yeasts and molds count (TYMC), and the specific presence of *Staphylococcus aureus*, *Pseudomonas aeruginosa*, *Clostridium* spp., and *Candida albicans* in the samples. Enumeration tests for all samples followed the guidelines outlined in 〈61〉 Microbiological examination of nonsterile products: Microbial enumeration tests, as per the United States Pharmacopoeia 43 and National Formulary 38 (USP 43-NF 38) [28].

### 2.3. Formulation and Evaluation of Emulsion Containing Ostrich Oil

#### 2.3.1. Formulation of Emulsion

In emulsification, phase inversion is induced by various parameters such as temperature, salt concentration, oil or water fraction, and energy input. For the preparation of O/W emulsions, the conventional method involves introducing the oil phase to the aqueous phase. However, in this study, we chose to employ the phase inversion technique, wherein the aqueous phase is added to the oil phase. This decision was motivated by reports indicating that emulsions formed using this technique contain a finely dispersed internal phase, contributing to their stability [29]. In this study, the phase inversion of an ostrich oil–lecithin–water emulsion system might be triggered by altering the water volume fraction or temperature. Additionally, W/O emulsions can be converted to O/W systems by incorporating lecithin, a hydrophobic emulsifier. The research aimed to investigate the effects of ostrich oil and lecithin concentrations on the physicochemical properties of emulsions. Consequently, we examined the impact of ostrich oil concentrations ranging from 5% *w*/*w* to 30% *w*/*w* and lecithin concentrations ranging from 1% *w*/*w* to 15% *w*/*w* to determine the stable O/W emulsion with the maximum ostrich oil concentration. Table 1 presents formulations of 10% *w*/*w* ostrich oil emulsions stabilized with lecithin concentrations ranging from 1% *w*/*w* to 15% *w*/*w*, while Table 2 showcases formulations of 5% *w*/*w* to 30% *w*/*w* ostrich oil emulsions stabilized with 10% *w*/*w* lecithin.

Using lecithin as an emulsifier, the phase inversion process resulted in the dispersion of ostrich oil droplets within a continuous aqueous phase. The ostrich oil was blended with lecithin at 50 °C using a magnetic hotplate stirrer (C-MAG HS 4 IKA, IKA-Werke GmbH & CO. KG, Staufen, Germany). Distilled water, heated to 50 °C, was consistently added to the oil phase. Subsequently, the emulsion was stirred with a magnetic hotplate stirrer at 50 °C for 10 min. The determination of the emulsion type was accomplished through a dye solubility test. Finally, the physicochemical properties of all prepared emulsions were evaluated.

#### 2.3.2. Evaluation of Emulsion

##### Microscopic Examination

The morphology of oil droplets was examined using an Olympus optical microscope (CX41RF, Olympus Corporation, Tokyo, Japan). To achieve an optimal concentration (10% *v*/*v*), the emulsion was appropriately diluted with distilled water. The diluted sample was then placed on a glass slide and covered with a cover slip for observation of the oil droplet morphology.

For the dye solubility test, the emulsion was mixed with a water-soluble dye (amaranth) and observed under the microscope to determine whether the dye dissolved in the aqueous phase or remained in the oil phase.

##### Physical Characteristics

The physical characteristics, including color and appearance, as well as the creaming indices of the emulsions, were assessed through visual observation on days 1, 3, and 7 after preparation. The creaming index (CI) was calculated using Equation (1), where “S” represents the height of the serum layer, and “T” denotes the total height of the emulsion.

(1) $$\%\;\mathrm{Creaming}\;\mathrm{index}=\frac{S}{T}\times 100$$

##### Droplet Size

The size of oil droplets in the emulsion was determined using a laser scattering particle size distribution analyzer (LA950, Horiba Ltd., Kyoto, Japan). The sample was appropriately diluted with distilled water to achieve an optimal concentration. The median diameter, representing the cumulative particle diameter equivalent to 50%, was recorded for each sample. All measurements were conducted in triplicate.

##### Zeta Potential Value

The zeta potential of the emulsion droplets was measured with a zeta potential analyzer (ZetaPlus, Brookhaven Instruments Corporation, Holtsville, NY, USA). Prior to analysis, the emulsion was diluted to an optimal concentration with distilled water. The average and standard deviation of measurements were recorded in triplicate for each sample.

##### Viscosity

The viscosity of each emulsion was tracked using a Brookfield DV-III Ultra Programmable Rheometer (RVDV-III Ultra, Brookfield Engineering Laboratories, Inc., Middleborough, MA, USA) equipped with a spindle CPE-51 or CPE-40. All measurements were conducted at 25 °C and performed in triplicate.

### 2.4. Fabrication and Evaluation of Dry Emulsions Containing Ostrich Oil

#### 2.4.1. Fabrication of Dry Emulsions

For the formulation of dry emulsions, we specifically chose a liquid O/W emulsion that includes ostrich oil and possesses the most suitable properties. Employing the modified adsorption technique, dry emulsions were meticulously prepared. The influence of different adsorbent types, such as Avicel^®^ PH-101 and Aerosil^®^ 200, on the properties of the dry emulsions was systematically evaluated. Each liquid O/W emulsion, containing ostrich oil, was individually mixed with every adsorbent using a planetary mixer until it reached an almost dry state. The resulting adsorbed emulsion was then sieved through a number 18 sieve and dried using a hot air oven at 50 °C for 6 h. Following the drying process, each obtained dry emulsion underwent another sieving stage through a number 20 sieve, and their respective properties were thoroughly assessed. Ultimately, the optimal dry emulsion was loaded into hard shell capsules (capsule number 0) using a capsule filling machine (Yeo Heng Co., Ltd., Pathum Thani, Thailand).

#### 2.4.2. Evaluation of Dry Emulsions

##### Physical Characteristics

The physical characteristics, such as appearance and color, of the dry emulsions were assessed in triplicate through visual observation and measured using a colorimeter (FRU WF32, SciLution, Shenzhen, China). The CIE L* a* b* (CIELAB) color scale, rooted in the opponent-color theory, was employed. Within this scale, L* signifies lightness, a* represents the red/green coordinate (+a* indicating redness, and −a* indicating greenness), b* denotes the yellow/blue coordinate (+b* indicating yellowness, and −b* indicating blueness), while Δ*E* measures the color difference between two colors. The magnitude of color variation, categorized by Δ*E*, is as follows: indistinguishable difference (0–0.5); slight difference (0.5–1.5); noticeable difference (1.5–3.0); appreciable difference (3.0–6.0); large difference (6.0–12.0); and very obvious difference (>12.0).

##### Percentage of Moisture

Each dry emulsion was precisely weighed to approximately 1.0 g and then analyzed using a Sartorius moisture analyzer (Mettler-Toledo International, Inc., Göttingen, Germany). All measurements were carried out in triplicate for accuracy.

##### Percentage of Weight Loss after Oil Release

The dry emulsion, precisely weighed at 5.0 g, was introduced into a test tube. Subsequently, the dry emulsion was reconstituted with distilled water to the same volume as the initial liquid emulsion, followed by vortexing at a consistent time and speed. The resulting mixture underwent centrifugation using a Universal 320R centrifuge machine (Hettich, Kirchlengern, Germany) at 6000 rpm for 5 min to separate the sediment from the supernatant. The sediment was subsequently oven-dried at 50 °C overnight until a constant weight was achieved. The percentage of weight loss was determined using Equation (2):

(2) $$\%\;\mathrm{weight}\;\mathrm{loss}=\frac{\left(\mathrm{Initial}\;\mathrm{weight}-\mathrm{Remaining}\;\mathrm{weight}\right)}{\mathrm{Initial}\;\mathrm{weight}}\times 100$$

This method provides insight into the dry emulsion’s oil release characteristics, and the calculated percentage reflects the extent of weight loss due to the separation of oil from the dry emulsion.

##### Microbial Contamination

The investigation focused on microbial contamination, encompassing TAMC, TYMC, as well as the specific presence of *Salmonella* spp. and *Escherichia coli* in a 10 g dry emulsion sample. The determination of these contaminants was conducted using the spread plate technique, following the guidelines outlined in USP 43-NF 38 [28].

##### Heavy Metal Contamination

The concentrations of heavy metals in dry emulsions were assessed using ICP-MS, as detailed in Section 2.2.5. Heavy metal contents.

##### Particle Size Analysis

The particle sizes of dry emulsion granules were measured (*n* = 3) using an analytical sieve shaker (US standard sieve set, Retsch, West Germany). A granule sample (50 g) was placed on top of the sieve machine and shaken for 5 min. The granules remaining on each sieve in the stack were weighed, and the average particle size was calculated.

Additionally, dry emulsions were individually redispersed in distilled water. The size of oil droplets in the liquid emulsion was determined using a laser scattering particle size distribution analyzer (LA950, Horiba Ltd., Kyoto, Japan).

##### Compressibility Index

Based on 〈616〉 Bulk density and tapped density of powders, USP 43-NF 38 [30], a compressibility test was employed to anticipate granule flow characteristics. A granule sample (30 g) was deposited into a 100 mL cylinder and manually tapped 50 times from a height of 10 cm. The compressibility index was then calculated using Equation (3).

(3) $$\%\;\mathrm{Compressibility}=\frac{\left(\mathrm{Tapped}\;\mathrm{density}-\mathrm{Bulk}\;\mathrm{density}\right)}{\mathrm{Tapped}\;\mathrm{density}}\times 100$$

The tapped density represents the unsettled apparent volume, and bulk density is the final tapped volume. Bulk density and tapped density were determined by dividing the mass (g) by the respective volumes (mL) of granules in the cylinder before and after tapping.

##### Particle Morphology

The particle morphology of dry emulsions was assessed using a scanning electron microscopy-energy-dispersive X-ray spectrometer (SEM-EDS Mira3, TESCAN Brno s. r. o., Brno, Czech Republic). The granule sample was affixed to a metallic specimen (stub) and subsequently metallized (sputtered) with a thin layer of gold/palladium using a coater. Following metallization, the sample was then examined.

##### Disintegration

After loading dry emulsions into hard shell capsules (capsule number 0) with a capsule filling machine (Yeo Heng Co., Ltd., Pathum Thani, Thailand), the disintegration of the capsules was determined using a disintegration testing apparatus (Apparatus B basket-rack assembly, Erweka GmbH, Heusenstamm, Germany) following the test method outlined in 〈701〉 Disintegration, USP 43-NF 38 [31]. The capsule samples were placed in the baskets and immersed in chambers containing 0.05 M acetate buffer as the immersion fluid. The temperature was maintained at 37 ± 2 °C, and a device raised and lowered the basket in the immersion fluid at a rate of 29–32 cycles/min. The disintegration time of each capsule was recorded when visually observed to be completely disintegrated. All experiments were conducted (*n* = 6), and the mean and standard deviation were calculated.

### 2.5. Stability 

#### 2.5.1. Stability under Temperature Cycling

Amber bottles, each containing samples and sealed hermetically, underwent a stability assessment with a cycle of 24 h in a freezer at 4 ± 0.1 °C, followed by 24 h at 45 ± 0.1 °C and 75 ± 2% relative humidity (RH). The physicochemical properties were evaluated after six cycles of temperature cycling.

#### 2.5.2. Stability under Storage at Various Temperatures

The sample was divided into three portions, each placed in amber bottles and sealed hermetically. These bottles were then stored separately at different temperatures: 4 ± 0.1 °C, 25 ± 0.1 °C, and 45 ± 0.1 °C at 75 ± 2% RH. Physicochemical properties were assessed through triplicate measurements after 1, 3, and 6 months of storage.

#### 2.5.3. Color Intensity

The characteristic yellow color of ostrich oil was assessed using a U-2900 spectrophotometer (Hitachi, Tokyo, Japan). The sample was placed in a cuvette, and its absorbance was recorded at 425 nm. This method was employed to monitor changes in yellow color during stability testing. All samples were analyzed in triplicate.

### 2.6. Statistical Analysis

The data underwent statistical analysis employing the t-test and one-way analysis of variance (ANOVA) within version 16 of the SPSS program, based on triplicate measurements. Statistically significant results were considered for *p*-values less than 0.05.

## 3. Results

### 3.1. Preparation and Evaluation of Ostrich Oil

The yield of ostrich oil obtained from the low-temperature rendering method was 66.7%. The AV, PV, IV, and SV values were 0.1 ± 0.0 mg KOH/g of oil, 2.5 ± 0.1 mEq O_2_/kg of oil, 30.9 ± 1.0 g I_2_/100 g of oil, and 195.8 ± 3.9 mg KOH/g of oil, respectively. These values fall within the permissible limits set by the Codex Standard for Named Animal Fats (CODEX-STAN 211-1999), the Food and Agriculture Organization of the United Nations, and the World Health Organization (FAO/WHO) [32], which are 2.0 mg KOH/g of oil, 10 mEq O_2_/kg of oil, 36–47 g I_2_/100 g of oil, and 190–200 mg KOH/g of oil. The RI of the resulting ostrich oil was 1.4560 ± 0.0000 at 30 °C, closely aligning with the permissible limit (1.448–1.460). This similarity underscores the purity and authenticity of the ostrich oil sample. 

The results of ICP-MS revealed that the contents of arsenic (As) and iron (Fe) in the ostrich oil sample were 0.002 ± 0.001 mg/kg and 0.803 ± 0.148 mg/kg, respectively, falling within the permissible limits of 0.1 mg/kg and 1.5 mg/kg. Additionally, levels of copper (Cu) and lead (Pb) were undetected, meeting the allowable limits of 0.4 mg/kg and 0.1 mg/kg, as specified by the CODEX-STAN 211-1999, FAO/WHO [32]. The microbial limit test showed the absence of both TAMC and TYMC in the oil sample. Investigation into pathogens, including *S. aureus*, *P. aeruginosa*, *Clostridium* spp., and *C. albicans*, did not detect contamination in 1 g of any sample. These results signify that the prepared ostrich oil was of exceptional quality and deemed safe for human consumption.

The fatty acid composition of ostrich oil was analyzed using the GC-FID technique. The results indicated that the three most abundant fatty acids in ostrich oil were oleic acid (34.60 ± 0.01%), palmitic acid (28.42 ± 0.05%), and linoleic acid (27.73 ± 0.01%), followed by stearic acid (5.07 ± 0.05%), α-linolenic acid (3.02 ± 0.00%), myristic acid (0.93 ± 0.01%), and lauric acid (0.23 ± 0.01%), consistent with a previous report [5,6,19]. Evaluating the nutritional value of fats for human consumption commonly involves assessing the ratio of polyunsaturated fatty acids (PUFAs) to saturated fatty acids (SFAs). As per nutritional guidelines, the recommended PUFA/SFA ratio in the human diet should exceed 0.45 [33]. The oil sample’s PUFA/SFA ratio of 0.89 adheres to this nutritional recommendation.

The IC_50_ values, representing the efficiency of DPPH radical scavenging and copper-chelating activity of ostrich oil, were determined to be 39.92 ± 1.51 mg/mL and 23.15 ± 0.12 mg/mL, respectively. In comparison, Trolox demonstrated significantly lower IC_50_ values of 0.0043 ± 0.0001 mg/mL for DPPH radical scavenging, highlighting its superior antioxidant activity. Similarly, EDTA exhibited a markedly lower IC_50_ value of 1.0801 ± 0.0002 mg/mL for copper-chelating activity. A comparative statistical analysis (*p* < 0.05) confirmed that the differences in IC_50_ values between ostrich oil and the standard antioxidants (Trolox and EDTA) were statistically significant, emphasizing the relatively lower antioxidant and copper-chelating efficiency of ostrich oil. These findings align with a previous study [19] and underscore the notable antioxidant and metal-chelating potential of ostrich oil, positioning it as a promising natural source for health-promoting compounds.

The thorough examination of the prepared ostrich oil, encompassing assessments of physicochemical properties, heavy metal contents, microbial contamination, antioxidant activities, and fatty acid composition, in conjunction with its favorable PUFA/SFA ratio of 0.89, affirms its appropriateness for inclusion in dietary supplement formulations.

### 3.2. Formulation and Evaluation of Emulsion Containing Ostrich Oil

#### 3.2.1. Formulation of Emulsion

The research aimed to investigate the impact of ostrich oil and lecithin concentrations on emulsion properties. Initially, 10% *w*/*w* ostrich oil emulsions were formulated with lecithin concentrations ranging from 1% *w*/*w* to 15% *w*/*w* (Table 1). Subsequently, formulations were developed using 5% *w*/*w* to 30% *w*/*w* ostrich oil stabilized with 10% *w*/*w* lecithin (Table 2). Finally, assessments were conducted on emulsion appearance, % CI, viscosity, droplet size, and zeta potential. The results revealed that the O/W emulsion containing 10% *w*/*w* ostrich oil and 10% *w*/*w* lecithin exhibited the most homogeneous emulsion (0.00% CI over a 7-day period, as shown in Table 1 and Table 2) with the smallest particle size (3.93 ± 0.11 µm). A lower % CI indicates a more stable emulsion.

#### 3.2.2. Evaluation of Emulsion

##### Visual Observation and Creaming Indices

**Figure 1:**
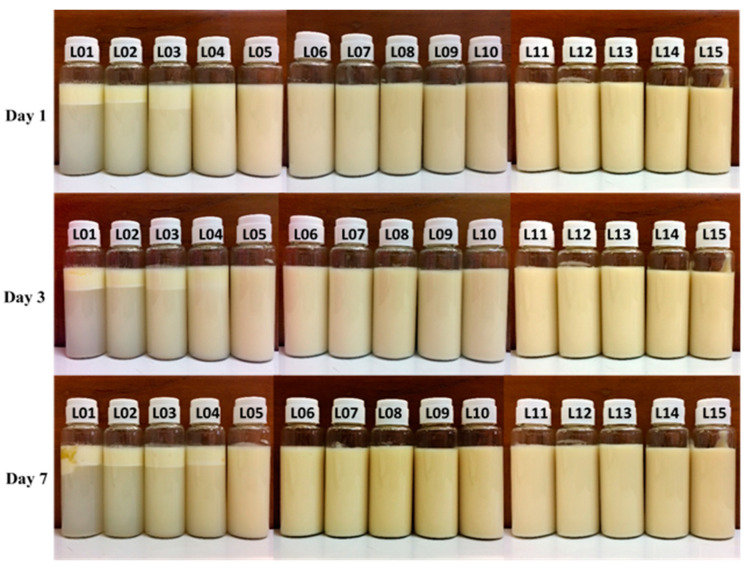
Appearances of the emulsions comprising 10% *w*/*w* ostrich oil and 1% *w*/*w* to 15% *w*/*w* lecithin on days 1, 3, and 7 (L01 to L15 represent lecithin concentrations ranging from 1% *w*/*w* to 15% *w*/*w*).

**Figure 2:**
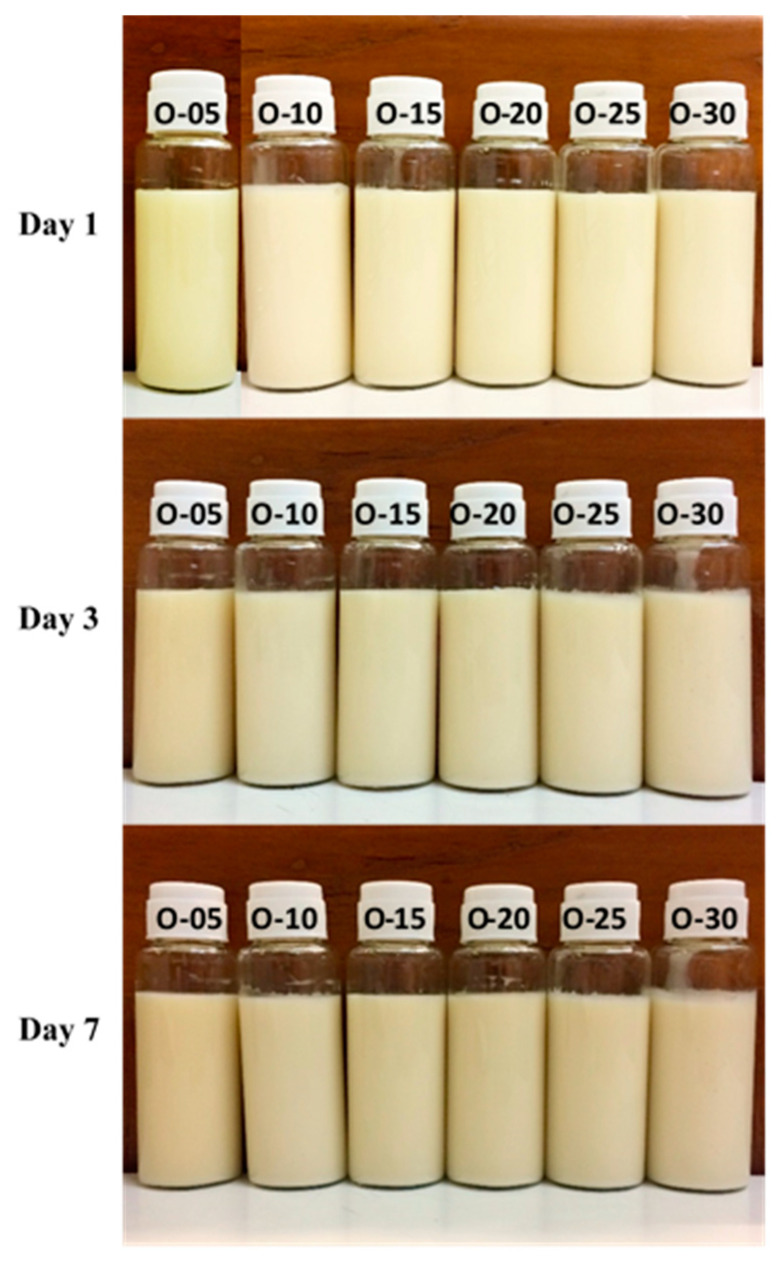
Appearances of the emulsions comprising 5% *w*/*w* to 30% *w*/*w* ostrich oil and 10% *w*/*w* lecithin on days 1, 3, and 7 (O-05 to O-30 represent ostrich oil concentrations ranging from 5% *w*/*w* to 30% *w*/*w*).

The emulsions, stabilized with lecithin, experienced a phase inversion from W/O to O/W by manipulating the volume ratio of the aqueous phase during the emulsion formulation. In this investigation, amaranth, a water-soluble red dye, was used in a dye solubility test. Microscopic observation during this test revealed the dye’s dissolution in the aqueous phase, indicated by a distinct red appearance in the continuous phase, strongly suggesting the formation of O/W emulsions. The emulsification process utilized concentrations ranging from 1% *w*/*w* to 15% *w*/*w* lecithin and 5% *w*/*w* to 30% *w*/*w* ostrich oil (Table 1 and Table 2). Figure 1 and Figure 2 visually presented the physical characteristics of all emulsions, while Table 1 and Table 2 provided the % CI on days 1, 3, and 7.

The results demonstrated that 10% *w*/*w* ostrich oil emulsions remained stable with no phase separation when the concentration of lecithin was increased from 5% *w*/*w* to 15% *w*/*w*, as illustrated in Figure 1. Notably, all these emulsions consistently exhibited CI of 0.00% over the 7-day period, as detailed in Table 1. Additionally, emulsions consisting of 10% *w*/*w* lecithin and varying concentrations of ostrich oil (ranging from 5% *w*/*w* to 30% *w*/*w*) were also found to be stable with no phase separation, as indicated by Figure 2. These emulsions maintained a steady 0.00% CI over the 7-day observation period, as presented in Table 2. The findings underscore the importance of lecithin concentration in influencing the stability of ostrich oil emulsions, providing valuable insights into the formulation’s impact on emulsion behavior over time.

For the formulation of emulsions, we implemented the phase inversion technique, a method commonly employed in the preparation of O/W and water-in-oil W/O emulsions. In the context of O/W emulsions, this technique involves reversing the conventional order of addition of the oil and water phases during emulsion formation. Traditionally, the oil phase is introduced into the aqueous phase. However, with the phase inversion technique, the aqueous phase is added to the oil phase. This inversion leads to the formation of stable O/W emulsions with smaller droplet sizes and enhanced stability [29]. The phase inversion technique results in a fine dispersion of the oil phase within the aqueous phase, producing emulsions characterized by a finely dispersed internal phase and improved stability. This technique is particularly useful when fine emulsions are desired, or when specific characteristics such as stability are critical for the application or formulation. The effectiveness of this method can be influenced by various factors, including the choice of emulsifiers, the composition of the oil and water phases, and the specific methods employed during the emulsification process [29]. Typically, the phase inversion technique is associated with emulsions having a low HLB value. Emulsifiers with low HLB values, being more lipophilic, have a greater affinity for oil. In the context of O/W emulsions, low HLB emulsifiers are selected to stabilize the oil droplets within the water phase. By adding the water phase to the oil phase, the emulsifier can efficiently surround the oil droplets, resulting in a stable emulsion [29,34].

##### Viscosity

**Figure 3:**
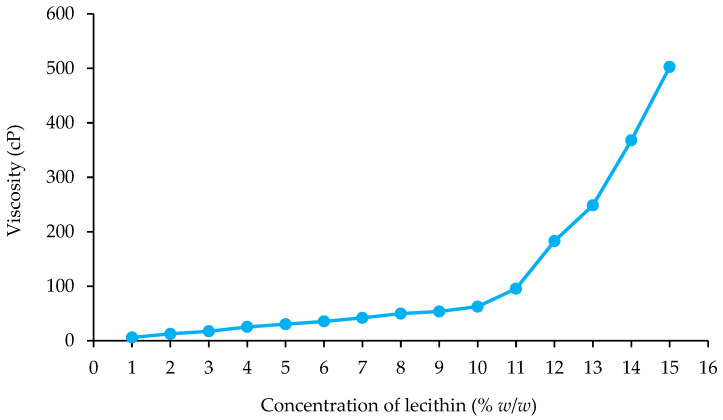
The viscosity of emulsions containing 10% *w*/*w* ostrich oil and 1% *w*/*w*–15% *w*/*w* lecithin.

**Figure 4:**
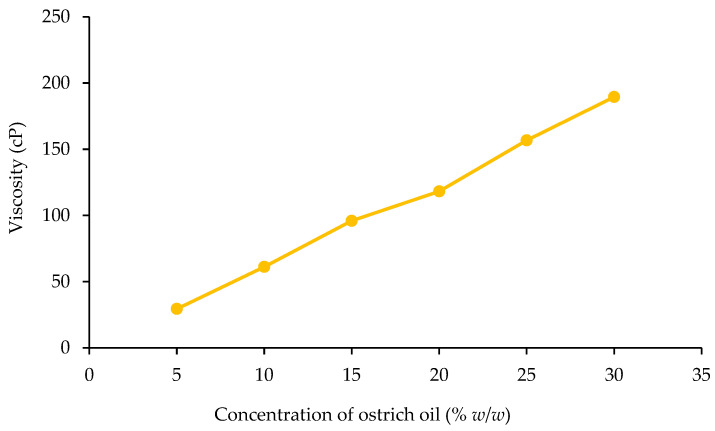
The viscosity of emulsions containing 5% *w*/*w*–30% *w*/*w* ostrich oil and 10% *w*/*w* lecithin.

The study delved into the viscosity characteristics of emulsions, investigating how varying concentrations of ostrich oil and lecithin influenced this property, as illustrated in Figure 3 and Figure 4. As the concentration of either ostrich oil or lecithin increased, there was a corresponding elevation in the viscosity of the emulsions. Notably, the viscosity of ostrich oil emulsions demonstrated a clear dependency on the concentrations of emulsifiers.

A discernible pattern emerged, indicating that higher emulsifier concentrations correlated with an increase in emulsion viscosity. This trend is attributed to the significant influence of emulsifier concentration on the stability of emulsions. Furthermore, the heightened viscosity of the continuous phase emerged as a crucial factor impacting droplet dynamics. The increased viscosity plays a pivotal role in impeding droplet mobility and discouraging droplet coalescence, thereby contributing significantly to the overall stability of the emulsion. This observation highlights the intricate relationship between emulsifier concentration, emulsion viscosity, and the resultant stability, providing valuable insights for optimizing emulsion formulations in future applications.

##### Droplet Size

**Figure 5:**
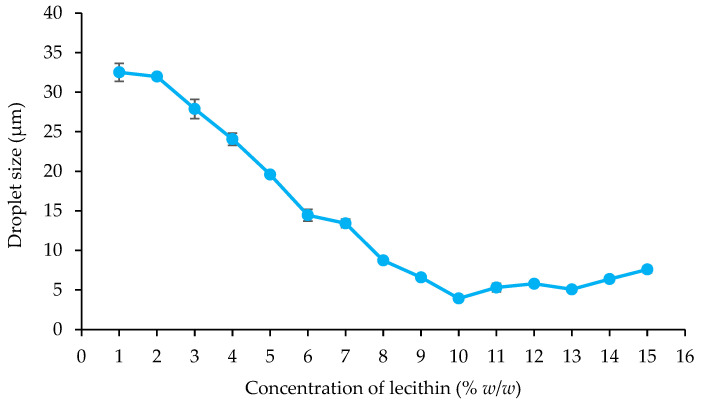
The droplet size of emulsions containing 10% *w*/*w* ostrich oil and 1% *w*/*w*–15% *w*/*w* lecithin.

Figure 5 illustrates the droplet size characteristics of emulsions composed of 10% *w*/*w* ostrich oil and lecithin concentrations ranging from 1% *w*/*w* to 15% *w*/*w*. An incremental increase in lecithin concentration, from 1% *w*/*w* to 10% *w*/*w*, resulted in a proportional reduction in the droplet size of the oil dispersion within the emulsions. This phenomenon is attributed to the heightened concentration of emulsifier effectively enveloping the oil droplets, leading to a decrease in interfacial tension among them, in accordance with a previous study [35]. At lecithin concentrations ranging from 10% *w*/*w* to 15% *w*/*w*, the droplet size remains relatively stable, with a slight minimum at 10% *w*/*w*, followed by a marginal increase as the concentration approaches 15% *w*/*w*. This behavior can be attributed to the complete coverage of the droplet surfaces by the emulsifier. Beyond 10% *w*/*w* lecithin, further increases in concentration do not significantly reduce droplet size. Moreover, the higher lecithin concentrations lead to increased viscosity in the continuous phase, which can hinder droplet deformation and contribute to the slight increase in size. These factors together explain the deviation from the trend observed at lower lecithin concentrations.

**Figure 6:**
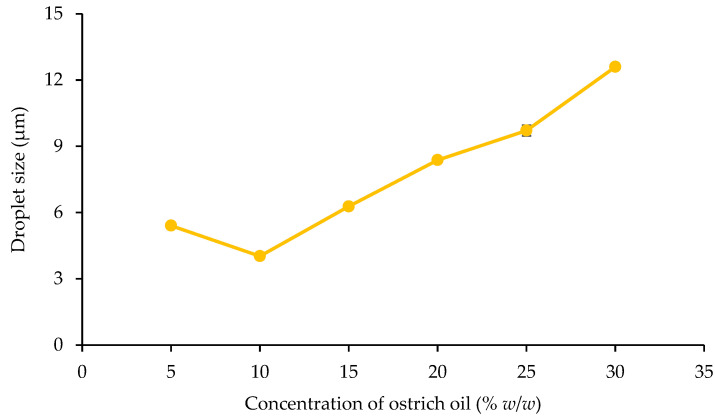
The droplet size of emulsions containing 5% *w*/*w*–30% *w*/*w* ostrich oil and 10% *w*/*w* lecithin.

Figure 6 shows the variation in droplet size for emulsions containing 10% *w*/*w* lecithin and 5% *w*/*w* to 30% *w*/*w* ostrich oil. The results indicate a significant increase in droplet size, particularly at concentrations above 15% *w*/*w*. This behavior can be attributed to the factors previously discussed in Figure 5, such as the coverage of emulsifier on the oil droplet surfaces and the increase in viscosity. At a lecithin concentration of 10% *w*/*w*, the high oil content may lead to incomplete coverage of the oil droplet surfaces, potentially causing coalescence. Additionally, the higher oil content increases the dispersed phase, raising the viscosity and reducing the emulsifier’s effectiveness, which contributes to the formation of larger droplets.

**Figure 7:**
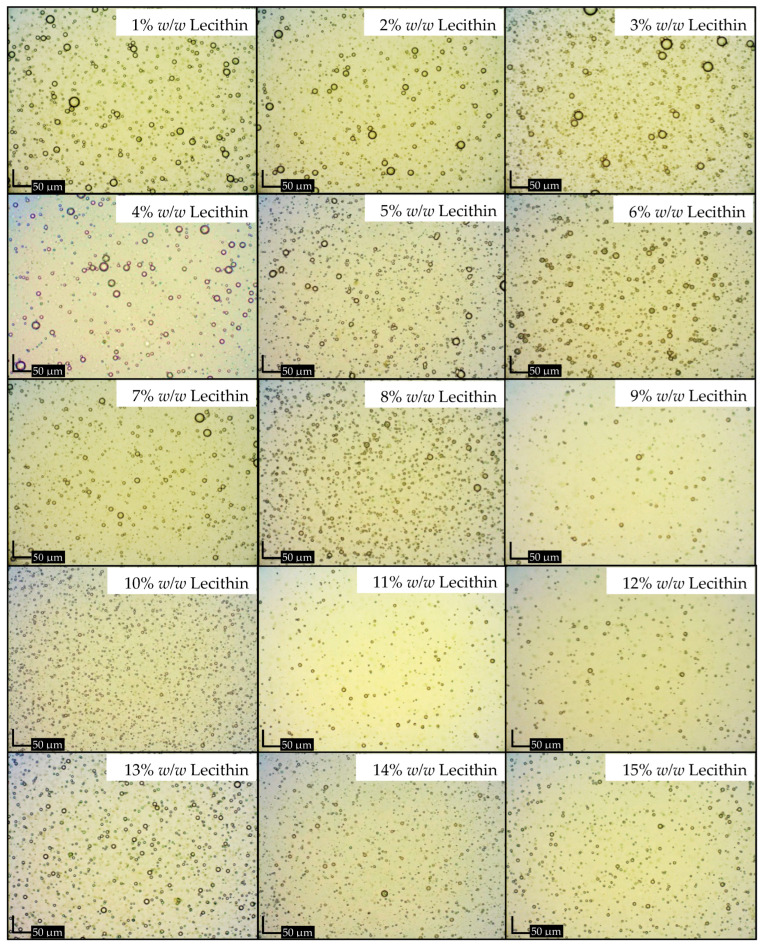
Photomicrographs of emulsions containing 10% *w*/*w* ostrich oil and 1% *w*/*w*–15% *w*/*w* lecithin.

**Figure 8:**
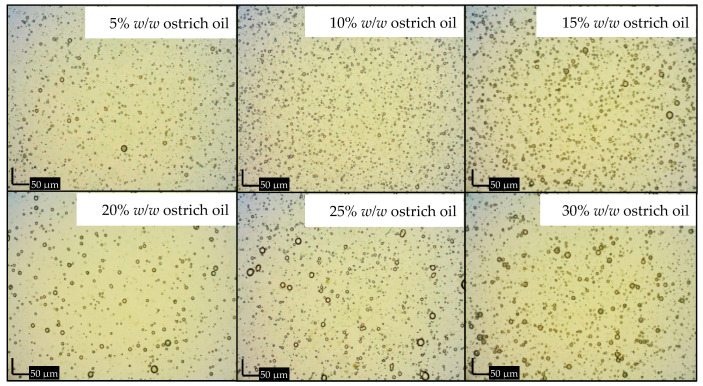
Photomicrographs of emulsions containing 5% *w*/*w*–30% *w*/*w* ostrich oil and 10% *w*/*w* lecithin.

Notably, the smallest droplet size (3.93 ± 0.11 μm) was observed in the emulsion containing 10% *w*/*w* ostrich oil, effectively stabilized with 10% *w*/*w* lecithin, aligning with photomicrographs of emulsions containing 10% *w*/*w* ostrich oil and 1–15% *w*/*w* lecithin (Figure 7 and Figure 8). The results indicated that the emulsion containing 10% *w*/*w* ostrich oil and 10% *w*/*w* lecithin had the smallest droplet size. Generally, the smallest droplet size signifies the most stable emulsions. These findings collectively suggest that emulsion formulations with 10% *w*/*w* concentrations of both ostrich oil and lecithin are optimal for achieving stable O/W emulsions, providing valuable insights for the formulation of dry emulsions.

##### Zeta Potential Value

**Figure 9:**
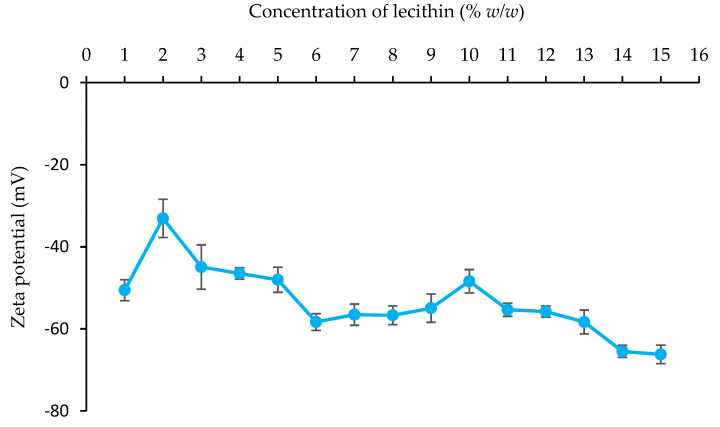
The zeta potential of emulsions containing 10% *w*/*w* ostrich oil and 1% *w*/*w*–15% *w*/*w* lecithin.

**Figure 10:**
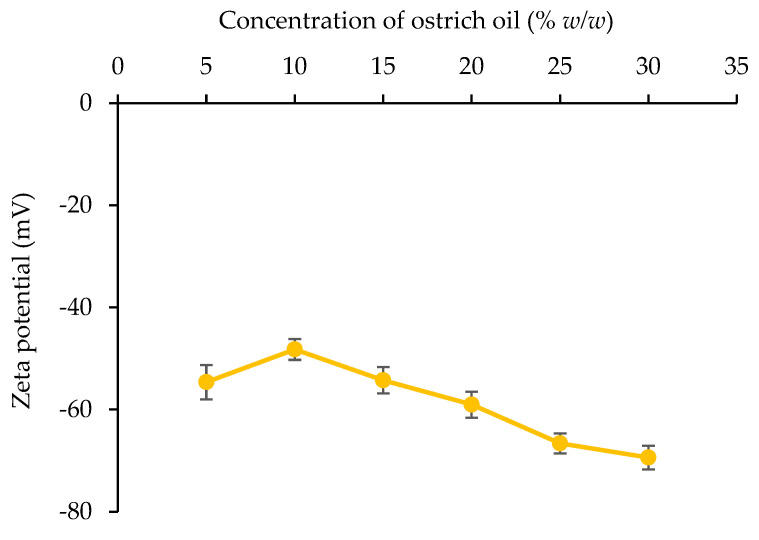
The zeta potential of emulsions containing 5% *w*/*w*–30% *w*/*w* ostrich oil and 10% *w*/*w* lecithin.

Figure 9 and Figure 10 present the zeta potential values of emulsions containing varying concentrations of lecithin and ostrich oil. Figure 9 specifically shows the zeta potential of emulsions with 10% *w*/*w* ostrich oil and lecithin concentrations ranging from 1% *w*/*w* to 15% *w*/*w*. All emulsions exhibit negative zeta potential values, primarily due to the negative charge of the phospholipid phosphate group in lecithin [36] and the carboxyl group of trace fatty acids [6] in ostrich oil at neutral pH. The zeta potential values fluctuate, generally decreasing as the lecithin concentration increases. At a low lecithin concentration of 1% *w*/*w*, the zeta potential is approximately −50 mV, increasing slightly to −33 mV as the lecithin concentration rises to 2% *w*/*w*. This increase may be attributed to the partial coverage of negatively charged oil droplets by lecithin at the surface, with the overall charge being the sum of both oil and lecithin. As the lecithin concentration exceeds 2% *w*/*w*, the zeta potential decreases further, reaching a minimum of −66 mV at 15% *w*/*w*. This decrease is likely due to improved stabilization of the droplets and more effective coverage of the oil–water interface by lecithin, leading to an enhanced negative surface charge.

Figure 10 presents the zeta potential of emulsions containing 10% *w*/*w* lecithin and ostrich oil concentrations ranging from 5% *w*/*w* to 30% *w*/*w*. As the ostrich oil concentration increases, the zeta potential shows a slight decrease. This trend can be attributed to the greater number of negatively charged oil droplets as the ostrich oil content rises. All emulsions exhibit very low zeta potential values (below −48 mV), indicating stable emulsions even with ostrich oil concentrations up to 30% *w*/*w*. These findings align with the creaming index (0% CI) observed for all emulsions, as shown in Table 2.

Emulsions characterized by significantly negative or positive zeta potentials typically demonstrate electrical stability, whereas those with lower absolute values of zeta potentials tend to be susceptible to flocculation and/or coagulation, potentially compromising stability. To ensure emulsion stability, zeta potential values for stable emulsions should exceed ±30 mV, thereby mitigating the risk of deflocculation within the emulsion system, in accordance with a previous study [37]. Particularly noteworthy is the emulsion formulation characterized by the smallest droplet size with a zeta potential exceeding −30 mV. Achieved through a precise blend of 10% *w*/*w* ostrich oil and 10% *w*/*w* lecithin, this formulation emerges as a promising candidate for initial emulsion development, holding potential for further advancement into a dry emulsion formulation containing ostrich oil.

### 3.3. Fabrication and Evaluation of Dry Emulsions Containing Ostrich Oil

#### 3.3.1. Fabrication of Dry Emulsions

In this investigation, an O/W emulsion was developed to enhance the stability of ostrich oil, an active ingredient with poor water solubility. The adsorption technique was employed for the formulation. The optimal liquid emulsion, comprising 10% *w*/*w* ostrich oil and 10% *w*/*w* lecithin, underwent transformation into a dry emulsion using adsorbents such as Avicel^®^ PH-101 (microcrystalline cellulose) and Aerosil^®^ 200 (colloidal silica). Avicel^®^ PH-101, a partially depolymerized cellulose with a particle size of approximately 50 μm, exhibits exceptionally high intraparticle porosity (90–95% of the surface area being internal). This porosity facilitates the swelling and disintegration of microcrystalline cellulose tablets due to water penetration into the hydrophilic tablet matrix, a process crucial for tablet dissolution. On the other hand, Aerosil^®^ 200 is a nonporous colloidal silicon dioxide classified as a hydrophilic adsorbent with a significant specific surface area of 200 m^2^/g [38]. Both Avicel^®^ PH-101 and Aerosil^®^ 200 are extensively employed in oral pharmaceutical formulations owing to their non-toxic nature. Furthermore, both substances have been included in the Generally Recognized as Safe (GRAS) list by the Food and Drug Administration (FDA) as food additives [39]. Given their established safety profiles, Avicel^®^ PH-101 and Aerosil^®^ 200 were selected as the preferred adsorbents for this study. By varying the weight ratios between the adsorbents and ostrich oil emulsion, the optimal ratios were determined: 50:50 (*w*/*w*) for Avicel^®^ PH-101 and 32:68 (*w*/*w*) for Aerosil^®^ 200. These results indicate that Aerosil^®^ 200 has a higher adsorption capacity compared to Avicel^®^ PH-101, which is consistent with its observed higher weight retention.

**Figure 11:**
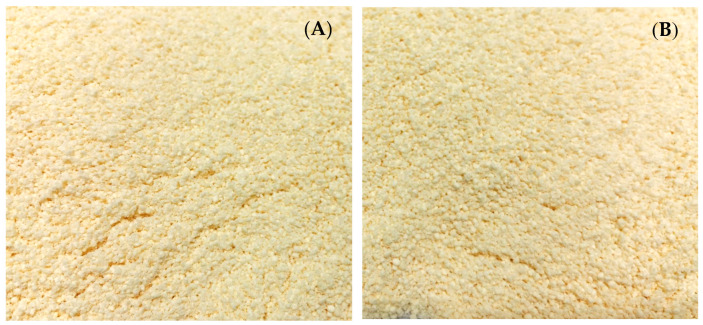
Appearances of the dry emulsions prepared using Avicel^®^ PH-101 (**A**) and Aerosil^®^ 200 (**B**) as adsorbents.

The visual representation of the dry emulsions prepared using Avicel^®^ PH-101 and Aerosil^®^ 200 can be observed in Figure 11. Results of color measurements for both granules, expressed by L*, a*, and b* components, are shown in Table 3. Both granules were very light (L* close to 100%) and had a slightly greenish-yellow color (negative a* and positive b*). It is noteworthy that the adsorption technique without heat employed for the formulation of both granules leads to lightening.

Optimal moisture control in dry emulsions is vital for ensuring stability, shelf life, and meeting quality standards, as excessive moisture can lead to issues like microbial growth, oxidation, and affect dispersibility and dissolution in liquid mediums. The moisture content of dry emulsions adsorbed with Avicel^®^ PH-101 (3.37 ± 0.07%) exceeded that of Aerosil^®^ 200 (2.00 ± 0.08%). Both dry emulsions demonstrated moisture contents within the general range (less than 3.5%) [40]. However, specific moisture content requirements may vary based on the formulation, type of adsorbent, and the drying process, as mentioned in earlier studies [13,40]. Therefore, understanding and tailoring moisture content to the specific needs of the formulation are imperative, ensuring that the dry emulsion meets quality standards and regulatory requirements.

**Figure 12:**
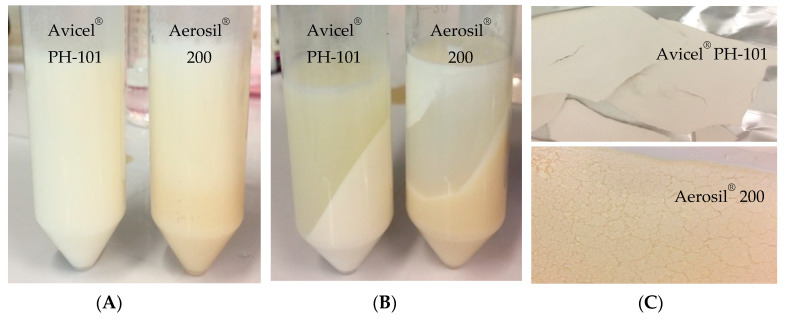
Visual observations of Avicel^®^ PH-101 and Aerosil^®^ 200 granules containing ostrich oil emulsion reconstituted with distilled water at room temperature before (**A**) and after (**B**) centrifugation, along with their dry sediments (**C**). The initial weight of the dry emulsion and the remaining weight (dry sediment) were used to calculate the percentage of weight loss after oil release.

To assess the liberation of ostrich oil from the granules, the percentage of weight loss following oil release was computed. The reconstituted emulsions derived from Avicel^®^ PH-101 and Aerosil^®^ 200 granules displayed a uniform appearance, mirroring their initial liquid emulsions containing ostrich oil (Figure 12A). Visual observations of both reconstituted emulsions post-centrifugation and their resulting dry sediment are presented in Figure 12B,C, respectively. The initial weight of the dry emulsion and the remaining weight (dry sediment) for each were utilized to calculate the percentage of weight loss after the release of oil for each dry emulsion. Notably, the percentage of weight loss after oil release from Avicel^®^ PH-101 granules (82.00 ± 1.00%) surpassed that of Aerosil^®^ 200 granules (30.00 ± 0.67%). This discrepancy underscores Avicel^®^ PH-101’s greater capacity to release ostrich oil emulsion compared to Aerosil^®^ 200 granules. The heightened hydrophobicity of Aerosil^®^ 200 relative to Avicel^®^ PH-101 resulted in a more efficient retention of ostrich oil, as previously established [40]. Despite Avicel^®^ PH-101’s lower adsorption capacity for ostrich oil emulsion compared to Aerosil^®^ 200, it demonstrated a superior capability to release the emulsion.

Ostrich oil emulsion, confined within a three-dimensional lattice of Aerosil^®^ 200 (colloidal silica), features a substantial proportion of liquid oil entrapped in a network of structuring molecules. This network immobilized the ostrich oil emulsion, transforming it into a material that maintained its integrity without leakage. The colloidal silica particle network, responsible for encapsulating the ostrich oil emulsion, was expected to impede its release due to the interactions between the oil and the particles, which reinforced their resistance to separation, as demonstrated in a previous study [41]. Consequently, Avicel^®^ PH-101 was selected as the preferred adsorbent for formulating dry emulsions of ostrich oil.

**Figure 13:**
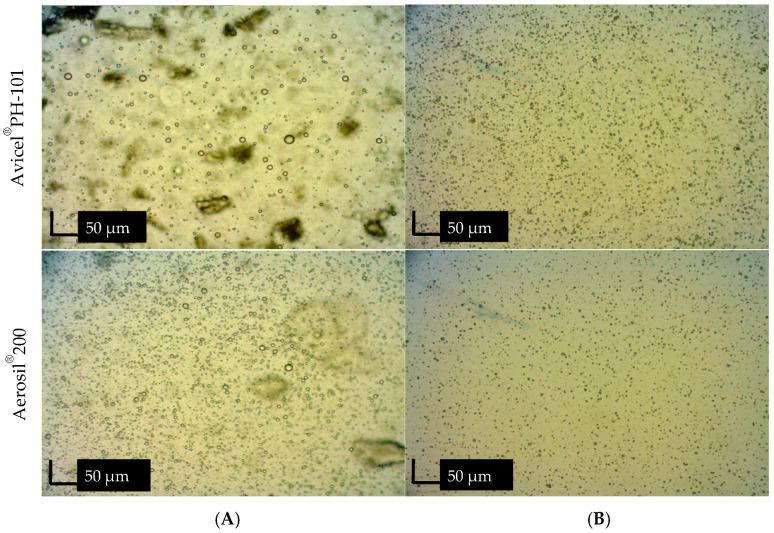
Photomicrographs of Avicel^®^ PH-101 (**upper**) and Aerosil^®^ 200 (**lower**) granules containing ostrich oil emulsion reconstituted with distilled water at room temperature before (**A**) and after (**B**) centrifugation.

To confirm the homogeneity of both reconstituted emulsions, Figure 12A presents visual observations of Avicel^®^ PH-101 and Aerosil^®^ 200 granules containing ostrich oil emulsion, reconstituted with distilled water at room temperature. The observations indicate that the mixtures are homogeneous. Figure 13 displays photomicrographs of the reconstituted emulsions obtained from Avicel^®^ PH-101 and Aerosil^®^ 200 granules, reconstituted with distilled water at room temperature, both before (Figure 13A) and after (Figure 13B) centrifugation. In Figure 13B, the photomicrographs depict the liquid emulsions after the adsorbents (Avicel^®^ PH-101 and Aerosil^®^ 200) have been separated from the reconstituted emulsions through centrifugation. The upper layer of the separated liquid emulsions appears homogeneous, with no signs of flocculation or coalescence. This observation suggests that the reconstituted emulsions maintain their stability and uniformity even after the removal of the adsorbents. The homogeneity of the liquid emulsions post-centrifugation indicates that the original dry emulsions were well-formulated and stable. If the emulsions had shown any signs of instability, such as phase separation or aggregation, it would have suggested potential issues with the stability of the dry emulsions or their formulation. The absence of flocculation or coalescence in the reconstituted emulsions confirms that the dry emulsions were effectively stabilized and that the reconstitution process successfully maintained the desired properties of the liquid emulsions.

#### 3.3.2. Evaluation of Avicel^®^ PH-101 Granules Containing Ostrich Oil Emulsion

Due to its superior capability to release the emulsion, the dry emulsion in the form of granules, prepared from a liquid emulsion containing 10% *w*/*w* ostrich oil and 10% *w*/*w* lecithin with Avicel^®^ PH-101 as an adsorbent using the adsorption technique, was evaluated for physical properties, heavy metal contents, and microbial contamination. The particle size of the granules was primarily measured at 401.50 ± 1.57 µm. The compressibility index provides insights into bulk density and anticipated powder flow behavior. For Avicel^®^ PH-101 granules containing ostrich oil emulsion, the compressibility index was found to be 19.43 ± 0.95%. This outcome classified the flowability of the granules as fair, in accordance with the flowability scale outlined in 〈1174〉 Powder flow, USP 43-NF 38 [42]. The classification of flowability as fair suggested that the granules exhibited moderate flow properties.

The complete dissolution of all capsules, each containing these granules (*n* = 6), occurred during a 30 min immersion period, in accordance with the specifications outlined in 〈2040〉 Disintegration and dissolution of dietary supplements, USP 43-NF 38 [43]. The calculated mean disintegration time, at 3.11 ± 0.14 min, further supports the efficient breakdown of the granules, indicating their suitability for oral administration and absorption of the encapsulated ostrich oil emulsion. These results collectively contribute to the overall assessment of the granules’ physical characteristics, emphasizing their potential as a viable formulation for delivering the ostrich oil emulsion in dietary supplements.

The levels of heavy metals in Avicel^®^ PH-101 granules containing ostrich oil emulsion were determined to be within the allowed thresholds, with arsenic (As) < 0.0005 mg/kg, cadmium (Cd) < 0.0005 mg/kg, lead (Pb) = 0.0860 mg/kg, and mercury (Hg) = 0.0007 mg/kg. These values comply with the established limits for element contaminants in dietary supplements, as stipulated by the guidelines in 〈2232〉 Element contaminants in dietary supplements, USP 43-NF 38 (As < 1.5 mg/kg, Cd < 0.5 mg/kg, Pb < 0.5 mg/kg, and Hg < 1.5 mg/kg) [44]. Regarding microbial attributes, the microbial counts in the Avicel^®^ PH-101 granules containing ostrich oil emulsion adhered to the defined microbial limit standards outlined for nonsterile nutritional and dietary supplements in 〈2023〉 Microbiological attributes of nonsterile nutritional and dietary supplements, USP 43-NF 38 [45]. Both the TAMC and TYMC were below 10^4^ cfu/g and 10^3^ cfu/g, respectively. Notably, *Salmonella* spp. and *E. coli* were not detected in the sample (10 g). The results of heavy metal contents and microbial loads affirmed the safety and compliance of the formulated dry emulsion granules with established quality and regulatory standards for dietary supplements.

**Figure 14:**
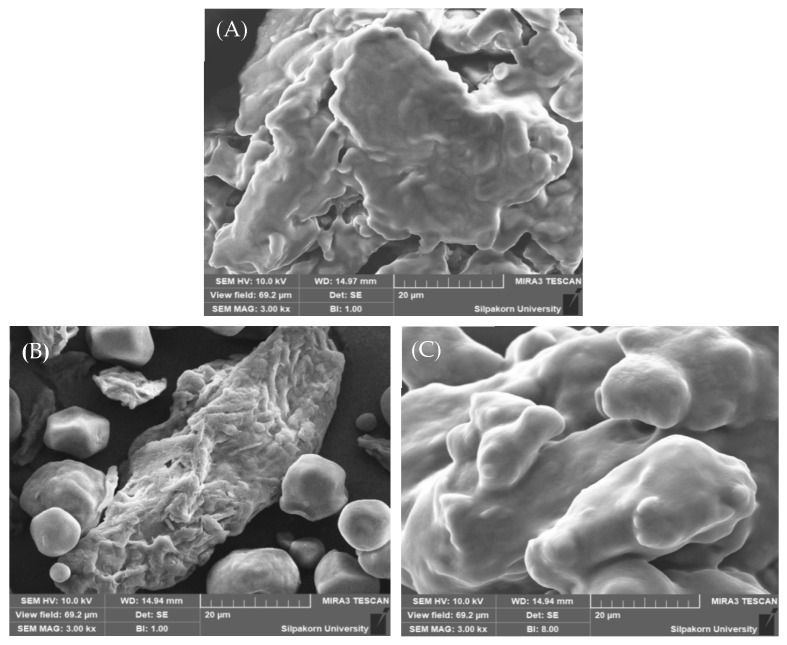
Scanning electron microscopy (SEM) images of Avicel^®^ PH-101 granules containing ostrich oil emulsion (**A**), Avicel^®^ PH-101 (**B**), and lecithin (**C**).

The SEM images (Figure 14) offer valuable insights into the surface morphology of the formulated granules. Notably, Avicel^®^ PH-101 granules containing ostrich oil emulsion appeared smoother compared to Avicel^®^ PH-101 alone, indicating a more uniform texture. However, it is important to note that, in comparison to pure lecithin, the surface of the Avicel^®^ PH-101 granules containing ostrich oil emulsion still exhibited a relatively rougher texture. This difference suggests interactions between Avicel^®^ PH-101 and the emulsion components, influencing granule topography. These observations highlight the impact of ostrich oil emulsion incorporation on granule surface morphology, providing valuable insights for understanding the physical attributes of the granules.

**Figure 15:**
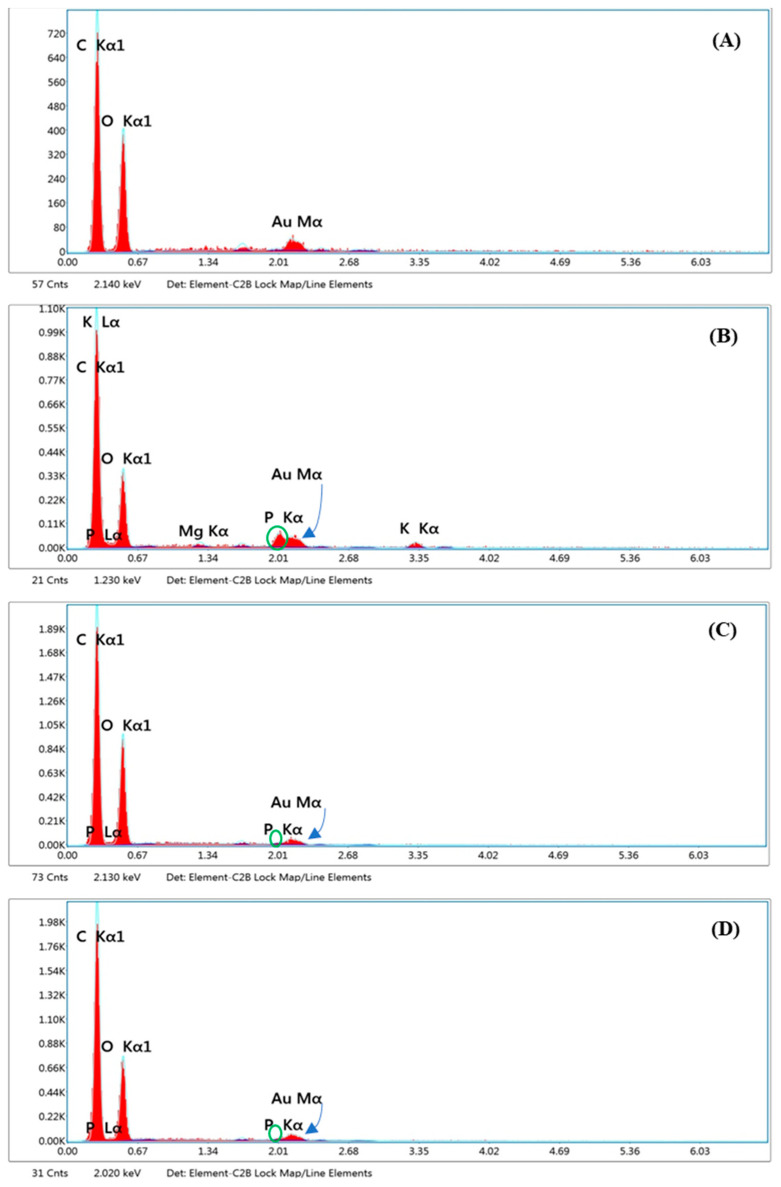
Scanning electron microscopy-energy-dispersive X-ray spectroscopy (SEM-EDS) images of Avicel^®^ PH-101 (**A**), lecithin (**B**), and Avicel^®^ PH-101 granules containing ostrich oil emulsion stored at 4 °C (**C**) and 45 °C (**D**), RH 75 ± 2%. The peak at 2.01 keV (indicated by the green circle) corresponds to the phosphate element, while the peak at 2.12 keV (marked by the blue arrow) is attributed to the gold element.

The SEM-EDS has yielded valuable insights into the interaction between Avicel^®^ PH-101 granules and the ostrich oil emulsion, utilizing lecithin as an emulsifier. The analysis uncovered a distinctive phosphate peak in the spectrum corresponding to lecithin (Figure 15B). Notably, this phosphate peak exhibited a significant decrease in the spectrum obtained from Avicel^®^ PH-101 granules containing the ostrich oil emulsion (Figure 15C,D). The reduction in the phosphate peak in the presence of the ostrich oil emulsion strongly indicates the loading of the ostrich oil emulsion, emulsified with lecithin, onto the Avicel^®^ PH-101 adsorbent. Avicel^®^ PH-101, recognized for its high adsorption capacity, appears to efficiently adsorb and encapsulate the ostrich oil emulsion, resulting in a modification of the elemental composition observed through SEM-EDS analysis. Furthermore, Avicel^®^ PH-101 granules containing ostrich oil emulsion, stored at 4 °C (Figure 15C) and 45 °C (Figure 15D), exhibited a decrease in phosphate peaks in the spectra, confirming the stability of the dry emulsion. This collective evidence reinforces the proposition that Avicel^®^ PH-101 granules serve as an efficient adsorbent for ostrich oil emulsion.

### 3.4. Stability

#### 3.4.1. Color and Morphological Stability

To assess physicochemical stability, ostrich oil and Avicel^®^ PH-101 granules containing ostrich oil emulsion, both with and without 0.01% *w*/*w* butylated hydroxytoluene (BHT), were subjected to alternating cycles of 24 h at 4 °C and 24 h at 45 °C for six cycles at 75 ± 2% RH. Prolonged stability testing was also conducted at 4 °C, 25 °C, and 45 °C for 180 days under consistent RH. UV–vis spectrophotometry at 425 nm was used to assess the stability of ostrich oil and ostrich oil with 0.1% *w*/*w* BHT. This analytical technique is effective for monitoring the stability of oil components, as their deterioration often corresponds to changes in the color profile of the oil [46]. The results showed that ostrich oil with BHT had lower absorbance compared to the oil without BHT, indicating enhanced stability. Over 180 days, samples without BHT at 45 °C had the highest absorbance, followed by those at 25 °C, and the lowest at 4 °C. Samples with BHT followed the same trend. From day 90 to day 180, ostrich oil with BHT stored at 45 °C showed similar absorbance to oil without BHT at 25 °C, while samples at 4 °C and those with BHT at 25 °C had similar values. These results suggest that BHT effectively stabilizes ostrich oil, especially at higher temperatures.

**Figure 16:**
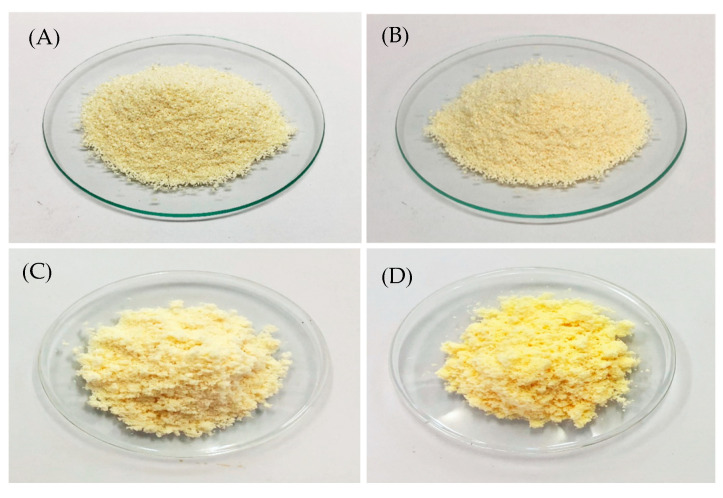
Visual aspects of Avicel^®^ PH-101 granules containing ostrich oil emulsion at the onset of storage (**A**) and following 180 days of storage at 4 °C (**B**), 25 °C (**C**), and 45 °C (**D**).

The impact of temperatures (4 °C, 25 °C, and 45 °C) on Avicel^®^ PH-101 granules containing ostrich oil emulsion was studied using optical microscopy and SEM analysis. Granules stored at 45 °C for 180 days exhibited the most pronounced yellow coloration, while those stored at 4 °C and 25 °C appeared pale yellow, as shown in Figure 16. The deepening yellow shade during extended storage at elevated temperatures is attributed to oxidation, indicating reduced stability of the granules. This discoloration highlights the sensitivity of the granules to oxidative degradation and the importance of temperature control for maintaining stability.

The color of Avicel^®^ PH-101 granules containing ostrich oil emulsion, with and without BHT, was assessed during stability testing with six cycles of temperature changes. Initial L*, a*, and b* values were around 92, −0.6, and 18, respectively, indicating high brightness. Color differences (Δ*E*) between granules with BHT (Δ*E* = 0.15) and without BHT (Δ*E* = 0.19) during cycling were indistinguishable. These results, detailed in Table 4, suggest that the granules maintain color consistency and stability under the specified conditions.

The study on the stability of Avicel^®^ PH-101 granules containing ostrich oil emulsion, with and without BHT, revealed the impact of storage conditions on color changes and surface morphology over 180 days. Table 5 presents color changes of granules stored at 4 °C, 25 °C, and 45 °C on days 0, 30, 90, and 180. Granules with BHT showed imperceptible color changes (Δ*E* 0.18 to 0.38) at 4 °C and 25 °C. However, at 45 °C, Δ*E* values increased to 1.64, 4.18, and 7.46 on days 30, 90, and 180, indicating noticeable to large color differences. The granules’ color shifted from pale yellow to intense yellow, highlighting temperature-dependent color stability.

For Avicel^®^ PH-101 granules containing ostrich oil emulsion without BHT, a slight color change (Δ*E* = 0.54) was observed at 25 °C for 180 days. In contrast, granules stored at 45 °C for 180 days exhibited a significant color change (Δ*E* = 19.97), as detailed in Table 5. A comparative statistical analysis (*p* < 0.05) confirmed that the color change at 45 °C was significantly greater than at 25 °C, underscoring the impact of temperature on granule stability. The addition of BHT provided significant protection against degradation at higher temperatures (*p* < 0.05), which is crucial for formulating and maintaining products with Avicel^®^ PH-101 granules and ostrich oil emulsion, ensuring long-term quality.

The particle size of Avicel^®^ PH-101 granules containing ostrich oil emulsion (401.50 ± 1.57 µm) did not change when stored at 4 °C and 25 °C. However, storage at 45 °C showed a tendency for particle agglomeration, which may be due to oil decomposition.

**Figure 17:**
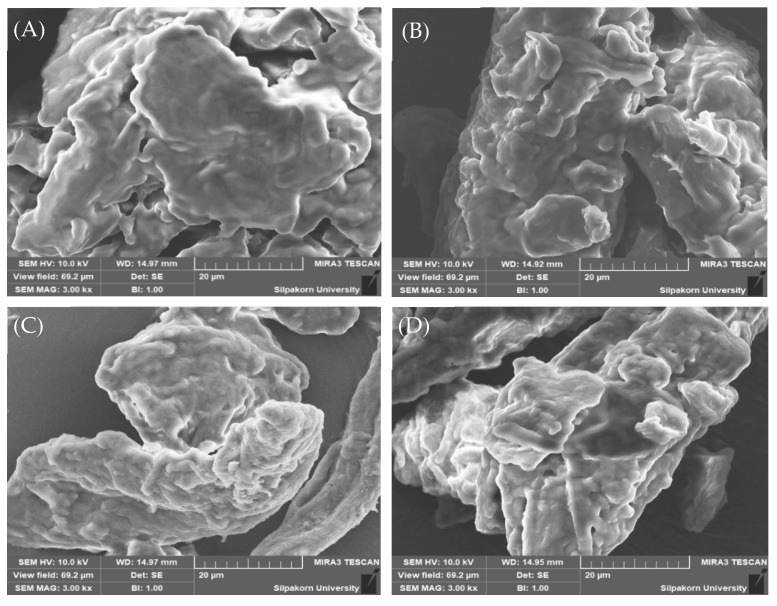
Scanning electron microscopy (SEM) images of Avicel^®^ PH-101 granules containing ostrich oil emulsion at the initial time (**A**) and after 180 days of storage at 4 °C (**B**), 25 °C (**C**), and 45 °C (**D**).

The SEM analysis of Avicel^®^ PH-101 granules with ostrich oil emulsion, performed initially and after 180 days at 4 °C, 25 °C, and 45 °C (Figure 17), showed consistent surface morphology across all conditions. This stability in surface appearance supports the color assessment findings and highlights the role of BHT in preserving granule integrity across various storage conditions.

#### 3.4.2. Physicochemical Stability

The physicochemical stability of ostrich oil and Avicel^®^ PH-101 granules, with and without BHT, was assessed using AV and PV. A high AV indicates triglyceride hydrolysis and rancidity, while PV measures initial oxidation through hydroperoxides. Under temperature cycling (6 cycles), ostrich oil without BHT exhibited the highest AV (0.11 ± 0.01 mg NaOH/g) and PV (1.50 ± 0.02 meq O_2_/kg). Avicel^®^ PH-101 granules containing ostrich oil emulsion without BHT also showed higher AV (0.09 ± 0.01 mg NaOH/g) and PV (1.10 ± 0.02 meq O_2_/kg) compared to those with BHT (AV = 0.08 ± 0.01 mg NaOH/g and PV = 0.95 ± 0.01 meq O_2_/kg).

As shown in Table 6, at elevated temperatures (25 °C and 45 °C), AV and PV increased, especially in samples without BHT. Ostrich oil with BHT showed the lowest AV and PV levels, while ostrich oil without BHT had lower values compared to Avicel^®^ PH-101 granules with ostrich oil emulsion without BHT. Notably, Avicel^®^ PH-101 granules with ostrich oil emulsion without BHT had the highest AV and PV readings, suggesting that lecithin and ostrich oil in the granules are prone to degradation and rancidity at high temperatures. These results highlight the need to carefully consider temperature-sensitive components in the formulation and storage of oil-based emulsions to prevent degradation.

In evaluating the quality of multi-ingredient dietary supplements as per USP 43-NF 38, AV and PV are analyzed for oil-based supplements. Although specific criteria for ostrich oil are lacking, this study uses the standards for fish oil and cod liver oil capsules [47,48]: AV should not exceed 3.0 mg NaOH/g, and PV should remain below 5.0 meq O_2_/kg. Freshly prepared Avicel^®^ PH-101 granules with and without BHT had AV and PV values of 0.08 ± 0.01 mg NaOH/g and 0.90 ± 0.01 meq O_2_/kg, respectively, well within these limits. Even after six cycles of stability testing and 180 days of storage at 4 °C and 25 °C, the AV and PV values remained below the criteria, with minimal impact from 4 °C storage. These results confirm that Avicel^®^ PH-101 granules with ostrich oil, with or without BHT, meet the guidelines of the Global Organization for EPA and DHA (GOED) [49,50], highlighting their safety and suitability for consumer use.

## 4. Discussion

### 4.1. Emulsion Formulation and Stability

This study investigated the impact of ostrich oil and lecithin concentrations on the stability and properties of emulsions. Our results demonstrate that the formulation with 10% *w*/*w* ostrich oil and 10% lecithin exhibited the best stability, characterized by a consistent 0.00% CI and the smallest droplet size of 3.93 ± 0.11 µm. These findings suggest that this formulation achieves an optimal balance between the oil and emulsifier concentrations, leading to enhanced emulsion stability. The effect of lecithin concentration on emulsion stability was significant. When lecithin concentration was varied from 1% to 15%, higher concentrations consistently improved stability by preventing phase separation. This improvement can be attributed to lecithin’s role as an emulsifier, which stabilizes the emulsion by forming a protective layer around the oil droplets. Notably, phase inversion from W/O to O/W was observed when the aqueous phase was adjusted, a transition that was effectively stabilized by the lecithin. This was further confirmed by the dye solubility test, which indicated successful stabilization of the emulsion. The observed phase inversion and enhanced stability are consistent with the known properties of lecithin, particularly its low HLB value. Lecithin’s ability to adapt to varying ratios of aqueous and oily phases contributes to its effectiveness in stabilizing emulsions and reducing droplet size. Additionally, increased lecithin concentrations led to higher viscosity, which aids in maintaining stability by reducing droplet mobility and preventing separation.

Our results also indicate that optimal stability was achieved with the 10% ostrich oil and 10% lecithin formulation. This formulation demonstrated the smallest droplet size and zeta potential values ranging from −33.08 ± 4.67 to −66.23 ± 2.25 mV. The negative zeta potential values suggest strong electrostatic repulsion between droplets, further contributing to emulsion stability. In conclusion, the formulation of 10% ostrich oil with 10% lecithin provides a robust and stable emulsion, highlighting its potential for further development into dry emulsions. The combination of optimal droplet size, high stability, and effective phase inversion indicates that this formulation can be effectively used in applications requiring stable emulsions with controlled properties.

### 4.2. Influence of Emulsifier Concentration

Soy lecithin is a versatile emulsifier known for its ability to stabilize O/W emulsions due to its unique HLB value. Its low HLB value enhances the stabilization of the oil phase, making it an effective choice for a wide range of applications, including food, pharmaceuticals, and cosmetics. The safety profile of soy lecithin further supports its broad use, as it is suitable for diverse age groups. In this study, we explored the influence of soy lecithin concentration on emulsion stability, employing the phase inversion technique to create stable O/W emulsions with optimal droplet sizes. Our findings indicate that increasing lecithin concentration significantly improved emulsion stability. Specifically, higher concentrations of soy lecithin led to increased viscosity of the emulsions, which in turn reduced droplet mobility and minimized coalescence. This is a critical observation, as reduced droplet mobility and coalescence directly contribute to enhanced emulsion stability. The results underscore the essential role of emulsifier concentration in determining the stability of emulsions. By increasing the lecithin concentration, we effectively enhanced the ability of the emulsifier to stabilize the oil phase and prevent separation, highlighting the importance of optimizing emulsifier levels for achieving desired emulsion properties.

Future research should consider additional variables that may affect emulsion performance, such as temperature, pH, and shear forces. Investigating these factors could provide further insights into how they interact with emulsifier concentration to refine and optimize emulsion formulations for specific applications. Understanding the complex interplay between these factors will be crucial for developing more effective and stable emulsions across various industries. Overall, this study demonstrates the significant impact of emulsifier concentration on emulsion stability and provides a foundation for further exploration of additional factors that influence emulsion performance.

### 4.3. Selection of Edible Adsorbents for Dry Emulsion

The preparation of dry emulsions involves careful selection of edible adsorbents to ensure effective stabilization and desirable release characteristics. In this study, Avicel^®^ PH-101 and Aerosil^®^ 200 were evaluated as potential adsorbents due to their distinct physicochemical properties and functionalities. Avicel^®^ PH-101, a microcrystalline cellulose with a particle size of 50 µm, specific surface area of 0.78 m^2^/g, and porosity of 40%, demonstrated notable advantages for dry emulsion applications. Its rapid water absorption and reduced sensitivity to water content make it particularly suitable for oral administration systems. The ability of Avicel^®^ PH-101 to enhance tablet hardness further supports its suitability for producing stable and effective dry emulsions. In contrast, Aerosil^®^ 200, a hydrophilic fumed silica with a finer particle size of 12 µm, a much larger specific surface area of 200 m^2^/g, and similar porosity of 40%, exhibited superior adsorption properties due to its high surface area and porous structure. This feature facilitates efficient liquid adsorption and stabilization during the drying process, which is crucial for maintaining the integrity of the emulsion.

Despite the advantages of Aerosil^®^ 200 in terms of adsorption capacity, Avicel^®^ PH-101 proved to be more effective in releasing the emulsion. The release efficiency of Avicel^®^ PH-101, combined with its disintegration time of 3.11 ± 0.14 min, underscores its practical utility in converting liquid emulsions into dry forms. This characteristic is particularly valuable for applications in dietary supplements where rapid dissolution and effective release are essential. The selection of Avicel^®^ PH-101 as the preferred adsorbent for dry emulsion formulation is based on its balanced performance, including efficient emulsion release and suitable disintegration time. While Aerosil^®^ 200 demonstrated superior adsorption, the overall performance of Avicel^®^ PH-101 in facilitating the conversion of liquid emulsions into dry forms justifies its choice for this application. The study highlights the importance of selecting the appropriate adsorbent based on specific application needs. Avicel^®^ PH-101’s effective release capabilities and disintegration performance make it a valuable choice for formulating dry emulsions, particularly for dietary supplements where both stability and rapid dissolution are critical. Future research could explore additional adsorbents and formulation techniques to further optimize dry emulsion systems for various applications.

### 4.4. Nutritional and Health Considerations of Ostrich Oil as a Dietary Supplement

Ostrich oil was selected for development into a food supplement due to its unique nutritional and therapeutic properties. It is rich in unsaturated fatty acids, particularly omega-3 and omega-6, and has a high antioxidant content, offering potential benefits for cardiovascular health, inflammation reduction, and overall wellness. Compared to common livestock and poultry fats, which are higher in saturated fats and lower in antioxidants, ostrich oil presents a superior profile for promoting health [51,52,53]. This makes it a promising candidate for enhancing dietary supplements. Ostrich oil is distinguished by its favorable fatty acid profile, which aligns closely with that of breast milk, a standard for essential fatty acid composition. Its PUFA-to-SFA ratio exceeds the recommended threshold of 0.45, indicating that it is a valuable source of essential fatty acids [33]. This ratio is crucial for supporting various health functions and adhering to dietary fat intake guidelines. The presence of both omega-6 and omega-3 fatty acids in ostrich oil contributes to its potential health benefits, including improved cardiovascular health and reduced risk of related diseases. Research has demonstrated that ostrich oil, when included in dietary products such as biscuits, does not adversely affect liver or kidney functions [54], suggesting that it is a safe addition to diets when used appropriately. Each capsule of ostrich oil dry emulsion provides significant amounts of omega-6 and omega-3 fatty acids, offering a potential health benefit. The beneficial effects of these fatty acids include supporting cardiovascular health and potentially reducing inflammation.

However, while the benefits of ostrich oil are notable, it is important to consider potential risks associated with its chronic consumption. There are concerns regarding hepatic injury and hypersensitivity with prolonged use, which necessitates a balanced evaluation of its long-term effects. This highlights the importance of conducting further research to thoroughly understand and validate the health benefits of ostrich oil as a dietary supplement. While ostrich oil demonstrates considerable promise as a source of essential fatty acids and a potential dietary supplement, it is crucial to approach its use with caution. Ongoing research is needed to establish optimal dosage strategies and confirm the long-term safety and efficacy of ostrich oil. Balancing its potential benefits with a thorough understanding of possible risks will ensure that it can be effectively and safely integrated into dietary regimens.

## 5. Conclusions

Ostrich oil was extracted from the abdominal adipose tissues of ostriches using a low-temperature wet rendering method. The resulting ostrich oil exhibited a pale-yellow color, with a yield of 66.7% from the extraction process. Its physicochemical properties, heavy metal levels, microbial counts, and fatty acid compositions adhered to the acceptable parameters outlined by the CODEX STAN 211-1999, FAO/WHO. Additionally, the ostrich oil exhibited noteworthy antioxidant activity and featured high concentrations of PUFAs. These findings collectively indicate that the ostrich oil obtained during the preparation process exhibits excellent quality, positioning it as a suitable candidate for subsequent development into an O/W emulsion and, ultimately, for formulation as a dry emulsion.

In addition to providing health benefits, lecithin maintains a high level of ADI compliance and was employed as an emulsifier for the formulation of the ostrich oil emulsion. The results indicated that the O/W emulsion, comprised of 10% *w*/*w* ostrich oil and 10% *w*/*w* lecithin prepared via phase inversion, manifested optimal viscosity (62.50 ± 1.01 cP), zeta potential (−48.40 ± 2.84 mV), and droplet size (3.93 ± 0.11 μm). Consequently, this formulation emerged as a suitable candidate for future development into a dry emulsion.

In the final section of this study, a dry emulsion incorporating ostrich oil emulsion was created using the adsorption technique to enhance the physicochemical stability of the ostrich oil emulsion. The effects of adsorbents, such as Avicel^®^ PH-101 and Aerosil^®^ 200, on the properties of granulated ostrich oil dry emulsion were studied. The findings indicated that Avicel^®^ PH-101 exhibited a more effective discharge of ostrich oil emulsion compared to Aerosil^®^ 200. The results affirm Avicel^®^ PH-101 as a suitable adsorbent for the formulation of ostrich oil dry emulsion.

In summary, the dry emulsion, composed of Avicel^®^ PH-101, ostrich oil, and lecithin, yielded favorable outcomes across all evaluation tests. The granule particle size averaged 401.50 ± 1.57 µm, exhibiting a moderately smooth flow. For easier swallowing, the granulated dry emulsion was filled into capsules. Disintegration time for all granule-filled capsules adhered to the USP 43-NF 38 criteria, with a mean of 3.11 ± 0.14 min. Both microbial loads and heavy metal contents remained within acceptable thresholds. The dry emulsion of ostrich oil, presented as granules containing BHT, showcased robust temperature stability along with promising attributes. These findings suggest potential applicability in the development of dietary supplements encompassing diverse animal oils.

## Figures and Tables

**Table 1:** Formulations of 10% *w*/*w* ostrich oil emulsions stabilized with 1% *w*/*w* to 15% *w*/*w* lecithin and their corresponding percent creaming indices (% CI) on days 1, 3, and 7.

Formulation	Lecithin (% *w*/*w*)	Distilled Water (% *w*/*w*)	Ostrich Oil (% *w*/*w*)	% Creaming Index (% CI)		
				Day 1	Day 3	Day 7
L01	1	89	10	78.38	78.38	77.78
L02	2	88	10	80.56	80.56	80.56
L03	3	87	10	77.78	75.68	72.22
L04	4	86	10	0.00	78.38	76.32
L05	5	85	10	0.00	0.00	0.00
L06	6	84	10	0.00	0.00	0.00
L07	7	83	10	0.00	0.00	0.00
L08	8	82	10	0.00	0.00	0.00
L09	9	81	10	0.00	0.00	0.00
L10	10	80	10	0.00	0.00	0.00
L11	11	79	10	0.00	0.00	0.00
L12	12	78	10	0.00	0.00	0.00
L13	13	77	10	0.00	0.00	0.00
L14	14	76	10	0.00	0.00	0.00
L15	15	75	10	0.00	0.00	0.00

**Table 2:** Formulations of 5% to 30% *w*/*w* ostrich oil emulsions stabilized with 10% *w*/*w* lecithin and their corresponding percent creaming indices (% CI) on days 1, 3, and 7.

Formulation	Lecithin (% *w*/*w*)	Distilled Water (% *w*/*w*)	Ostrich Oil (% *w*/*w*)	% Creaming Index (% CI)		
				Day 1	Day 3	Day 7
O-05	10	85	5	0.00	0.00	0.00
O-10	10	80	10	0.00	0.00	0.00
O-15	10	75	15	0.00	0.00	0.00
O-20	10	70	20	0.00	0.00	0.00
O-25	10	65	25	0.00	0.00	0.00
O-30	10	60	30	0.00	0.00	0.00

**Table 3:** Color components of the dry emulsions prepared using Avicel^®^ PH-101 and Aerosil^®^ 200.

Color Components	Dry Emulsions	
	Avicel^®^ PH-101	Aerosil^®^ 200
Granule color		
L*	92.48 ± 0.43	92.55 ± 0.37
a*	−0.32 ± 0.07	−0.47 ± 0.10
b*	18.44 ± 0.16	18.60 ± 0.12

**Table 4:** Color values (L*, a*, b*, and ∆*E*) of Avicel^®^ PH-101 granules containing ostrich oil emulsion with and without BHT exposed to temperature cycling for 6 cycles.

Avicel^®^ PH-101 Granules Containing Ostrich Oil Emulsion	Cycle	L*	a*	b*	∆*E*
With BHT	0	92.37 ± 0.47	−0.64 ± 0.17	18.32 ± 0.26	
	6	92.23 ± 0.27	−0.63 ± 0.10	18.38 ± 0.62	0.15
Without BHT	0	92.34 ± 0.43	−0.68 ± 0.10	18.29 ± 0.16	
	6	92.17 ± 0.45	−0.62 ± 0.07	18.24 ± 0.37	0.19

L* indicates lightness; a* is the red/green coordinate (+a* = redness, −a* = greenness); b* is the yellow/blue coordinate (+b* = yellowness, −b* = blueness); Δ*E* is the difference between two colors.

**Table 5:** Color values (L*, a*, b*, and ∆*E*) of Avicel^®^ PH-101 granules containing ostrich oil emulsion with and without BHT, stored at 4 °C, 25 °C, and 45 °C, on days 0, 30, 90, and 180.

Ostrich Oil—Avicel^®^101 Granules	Day	L*	a*	b*	∆*E*
With BHT Stored at 4 °C	0	92.37 ± 0.47	−0.67 ± 0.18	18.32 ± 0.26	
	30	92.37 ± 0.42	−0.64 ± 0.08	18.10 ± 0.24	0.22
	90	92.37 ± 0.39	−0.67 ± 0.11	18.10 ± 0.32	0.22
	180	92.37 ± 0.70	−0.63 ± 0.12	18.14 ± 0.21	0.18
With BHT Stored at 25 °C	0	92.37 ± 0.47	−0.64 ± 0.17	18.32 ± 0.26	
	30	92.30 ± 0.72	−0.67 ± 0.07	18.09 ± 0.19	0.24
	90	92.13 ± 0.16	−0.62 ± 0.03	18.06 ± 0.24	0.35
	180	92.00 ± 0.17	−0.62 ± 0.09	18.22 ± 0.11	0.38
With BHT Stored at 45 °C	0	92.37 ± 0.47	−0.64 ± 0.17	18.32 ± 0.26	
	30	91.66 ± 0.10	0.58 ± 0.07	19.15 ± 0.38	1.64
	90	89.88 ± 0.34	1.48 ± 0.13	20.92 ± 0.26	4.18
	180	88.51 ± 0.21	2.36 ± 0.17	23.95 ± 0.06	7.46
Without BHT Stored at 4 °C	0	92.34 ± 0.43	−0.68 ± 0.10	18.29 ± 0.16	
	30	92.31 ± 0.78	−0.64 ± 0.13	18.23 ± 0.32	0.08
	90	92.38 ± 0.17	−0.64 ± 0.16	18.10 ± 0.15	0.20
	180	92.09 ± 0.19	−0.62 ± 0.13	18.13 ± 0.07	0.30
Without BHT Stored at 25 °C	0	92.34 ± 0.43	−0.64 ± 0.11	18.29 ± 0.16	
	30	92.22 ± 0.69	−0.42 ± 0.14	18.10 ± 0.11	0.31
	90	91.96 ± 0.15	−0.48 ± 0.15	18.12 ± 0.26	0.45
	180	91.93 ± 0.08	−0.44 ± 0.17	18.01 ± 0.14	0.54
Without BHT Stored at 45 °C	0	92.34 ± 0.43	−0.64 ± 0.11	18.29 ± 0.16	
	30	91.07 ± 0.18	0.62 ± 0.45	21.38 ± 0.05	3.57
	90	87.76 ± 0.67	3.64 ± 0.17	27.55 ± 0.13	11.18
	180	83.13 ± 0.22	6.41 ± 0.06	34.55 ± 0.23	19.97

L* indicates lightness; a* is the red/green coordinate (+a* = redness, −a* = greenness); b* is the yellow/blue coordinate (+b* = yellowness, −b* = blueness); Δ*E* is the difference between two colors.

**Table 6:** Acid values (AV) and peroxide values (PV) of ostrich oil and Avicel^®^ PH-101 granules containing ostrich oil emulsion (dry emulsion), both with and without BHT, stored at 4 °C, 25 °C, and 45 °C for 180 days.

Sample	Storage Temperature (°C)	AV (mg NaOH/g Sample)				PV (mEq O_2_/kg Sample)			
		Initial	1 Month	3 Months	6 Months	Initial	1 Month	3 Months	6 Months
Dry emulsion with BHT	4 °C	0.08 ± 0.01	0.10 ± 0.01	0.09 ± 0.01	0.09 ± 0.01	0.90 ± 0.01	1.00 ± 0.01	1.00 ± 0.01	1.00 ± 0.01
	25 °C		0.10 ± 0.01	0.09 ± 0.01	0.08 ± 0.01		1.00 ± 0.01	1.10 ± 0.02	1.10 ± 0.02
	45 °C		0.10 ± 0.01	0.12 ± 0.01	0.19 ± 0.02		1.05 ± 0.01	1.53 ± 0.02	2.51 ± 0.02
Dry emulsion without BHT	4 °C	0.08 ± 0.01	0.10 ± 0.01	0.09 ± 0.01	0.09 ± 0.01	0.90 ± 0.01	1.01 ± 0.02	1.15 ± 0.01	1.80 ± 0.02
	25 °C		0.10 ± 0.01	0.11 ± 0.02	0.14 ± 0.02		1.01 ± 0.01	1.45 ± 0.02	2.80 ± 0.02
	45 °C		0.12 ± 0.02	0.14 ± 0.02	0.30 ± 0.02		1.60 ± 0.01	4.09 ± 0.02	5.82 ± 0.04
Ostrich oil With BHT	4 °C	0.07 ± 0.01	0.10 ± 0.01	0.09 ± 0.01	0.09 ± 0.01	0.80 ± 0.02	0.80 ± 0.01	0.80 ± 0.01	1.00 ± 0.01
	25 °C		0.10 ± 0.01	0.09 ± 0.02	0.08 ± 0.01		0.08 ± 0.01	1.05 ± 0.01	1.10 ± 0.02
	45 °C		0.10 ± 0.02	0.12 ± 0.01	0.15 ± 0.02		0.95 ± 0.02	1.40 ± 0.02	2.31 ± 0.03
Ostrich oil Without BHT	4 °C	0.07 ± 0.01	0.10 ± 0.01	0.09 ± 0.01	0.09 ± 0.01	0.80 ± 0.01	0.80 ± 0.01	1.00 ± 0.01	1.80 ± 0.01
	25 °C		0.10 ± 0.01	0.11 ± 0.02	0.14 ± 0.01		0.90 ± 0.02	1.10 ± 0.02	2.65 ± 0.01
	45 °C		0.12 ± 0.02	0.13 ± 0.01	0.22 ± 0.02		1.50 ± 0.01	2.24 ± 0.03	5.61 ± 0.01

## Data Availability

The original contributions presented in the study are included in the article, further inquiries can be directed to the corresponding author.
